# Study of protease-mediated processes initiating viral infection and cell–cell viral spreading of SARS-CoV-2

**DOI:** 10.1007/s00894-022-05206-8

**Published:** 2022-07-19

**Authors:** Thanawat Thaingtamtanha, Stephan A. Baeurle

**Affiliations:** Department of Chemistry and Biology, Universität Siegen, Adolf-Reichwein-Str. 2, 57076 Siegen, Germany

**Keywords:** SARS-CoV-2, Viral-cell entry, Cell–cell viral spreading, Furin cleavage, TMPRSS2 cleavage

## Abstract

Viral-cell entry and cell–cell viral spreading processes of SARS-CoV-2 are subjected to fast evolutionary optimization because of its worldwide spreading, requiring the need for new drug developments. However, this task is still challenging, because a detailed understanding of the underlying molecular processes, mediated by the key cellular proteases TMPRSS2 and furin, is still lacking. Here, we show by large-scale atomistic calculations that binding of the ACE2 cell receptor at one of the heteromers of the SARS-CoV-2 spike leads to a release of its furin cleavage site (S1/S2), enabling an enhanced furin binding, and that this latter process promotes the binding of TMPRSS2 through the release of the TMPRSS2 cleavage site (S2′) out of the ACE2-binding heteromer. Moreover, we find that, after proteolytic cleavage, improved furin binding causes that parts of the S2 subunit dissociate from the complex, suggesting that furin promotes the fusion of the S2 subunit with the cell membrane before transfer of the viral RNA.

**Graphical abstract:**

Here we show by computational means that binding of the ACE2-cell receptor at one of the heteromers of the SARS-CoV-2 spike leads to an enhanced binding of the protease furin, promoting the binding of the protease TMPRSS2. Moreover, we show that, after proteolytic cleavage, improved furin binding causes that parts of the heteromer dissociate from the spike.

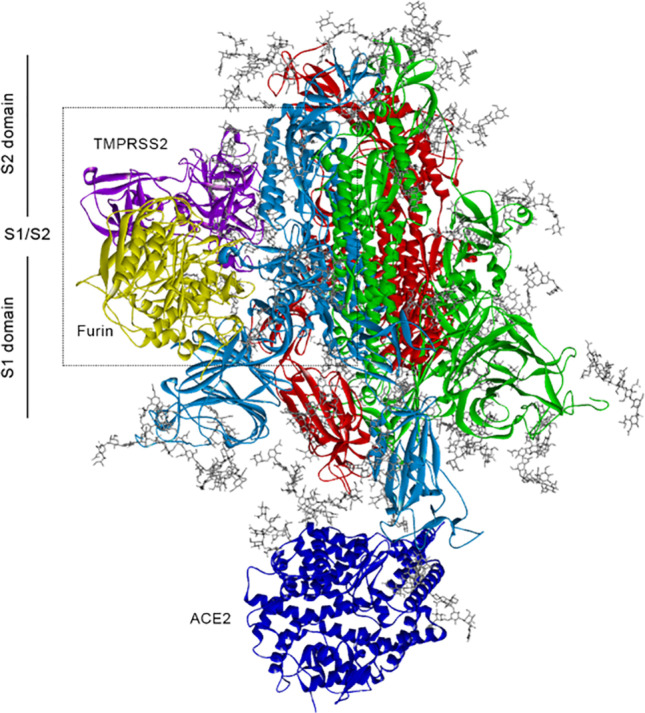

**Supplementary Information:**

The online version contains supplementary material available at 10.1007/s00894-022-05206-8.

## Introduction

The 2019 coronavirus disease (COVID-19) is a pandemic of major impact for human health and global economies [1]. It is caused by the severe acute respiratory syndrome coronavirus 2 (SARS-CoV-2), which belongs to the betacoronavirus family and is closely related to SARS-CoV-1, which was responsible for the epidemic in the year 2003 [2, 3]. Like for all coronaviruses, spikes are protruding from its viral surface, which are made of heterotrimers, where each unit comprises a glycoprotein consisting of ~ 1,300 amino acids [4, 5]. It is well established that the glycoproteins from such a surface glycoprotein trimer spike (S protein) can be cleaved by suitable proteases in different subunits, which possesses sites of different functionality [5]. For example, the N-terminal subunit S1 (~ 700 amino acids) contains the receptor binding domain (RBD) to the host cell, whereas the C-terminal subunit S2 (~ 600 amino acids) is membrane-anchored and harbors the fusion machinery [5]. Both subunits are known to play a decisive role during viral entry by recognizing host cell receptors, like, e.g., human angiotensin converting enzyme (ACE2), and mediating fusion of the viral and cellular membranes [5, 6]. Triggered by binding to the host cell receptor, proteolytic processing through cellular proteases and/or acidic pH in the cellular compartments, the S protein is known to undergo a transition from a metastable prefusion state to a stable postfusion state [5, 7]. Among the key cellular proteases for proteolytic processing of the S protein are furin and transmembrane serine proteinase 2 (TMPRSS2) [7]. Furin is a type I transmembrane serine protease that is ubiquitously expressed and cycles from the trans-Golgi network to the cell membrane, as well as through the endosomal system [8]. Proteolytic cleavage at the C-terminus of furin separates the transmembrane domain from the catalytically active domain. As a result, furin can be shed into the extracellular space as an active enzyme [9]. Furin proteolytically activates many precursor proteins [8]. Moreover, it is present in multiple organs and tissues in humans, such as the brain, lung, gastrointestinal tract, liver, pancreas, and reproductive tissues [10–12]. TMPRSS2 is a multidomain type II transmembrane serine protease that is expressed in humans in the prostate, colon, stomach, and salivary gland [13] as well as in specific cell types in the lungs and human bronchial epithelial cells [14]. Its physiological role is yet unknown [15].

Early works identified the existence of a minimal cleavage site motif (R-R-A-R685↓) at the boundary between the S1/S2 subunits of the heteromers in the S protein, which can be proteolytically processed by furin. Since it is lacking in SARS-CoV-1 and other SARS-related CoVs [12, 16, 17], they explained the increased tropism and higher transmissibility of SARS-CoV-2, compared to the other coronaviruses, by the more effective proteolytic cleavage at this furin cleavage site. Hoffmann et al. [18] showed that SARS-CoV-2 infection depends on the presence of the ACE2 receptor and TMPRSS2 in the cell membrane of lung cells, to cleave one of the heteromers of the S protein and allow viral entry. In a subsequent work [19], it was reported that furin cleaves the S protein at the S1/S2 site of a heteromer and that cleavage is essential for the SARS-CoV-2-S-protein-driven cell–cell fusion and entry into TMPRSS2^+^ lung cells. In addition, they found by optimizing the S1/S2 site that cell–cell fusion but not virus-cell fusion was increased and suggested from these results that the corresponding viral variants might exhibit increased cell–cell spread and potentially altered virulence. In a recent experimental study, Bestle et al. [15] showed that for viral entry of SARS-CoV-2 in human airway cells, one of the heteromers of a S protein has to be cleaved by furin and TMPRSS2 in a two-stage process. In the first step, it is cleaved proteolytically by furin into the subunits S1 and S2 at the S1/S2 cleavage site and in a subsequent step by TMPRSS2 at the S2′ site. TMPRSS2 can bind to the S2′ cleavage site, which is characterized by a paired dibasic motif with a single KR segment (KR815↓) [15], and can be found in SARS-CoV-2, SARS-CoV-1, and several other coronaviruses [16]. Cheng et al. [20] found that cleavage and the syncytium are abolished through treatment of VeroE6 cells with furin inhibitors decanoyl-RVKR-chloromethylketone (CMK) and naphthofluorescein, but not through treatment with the TMPRSS2 inhibitor camostat. They concluded from their results that cleavage of the furin cleavage site in the viral spike protein is critical for virus production and cytopathic effects. However, it is worth emphasizing that, to date, there is still no general consensus about the importance of the furin cleavage site for mediating the viral entry and cell–cell viral spread of SARS-CoV-2. For example, Walls et al. [10, 17] demonstrated in in vitro experiments with SARS-CoV-2 mutants, in which the furin cleavage site has been deleted, that these variants could still enter the cell lines of humans, African green monkeys, and bay hamsters. Xing et al*.* [10] deduced from the analysis of SARS-CoV-2-genome sequences with polymorphism at the furin cleavage site that it might not be required for SARS-CoV-2 to enter human cells in vivo and that the identified mutants may represent a new subgroup of SARS-CoV-2 coronaviruses with reduced tropism and transmissibility. Papa et al. [7] showed through CRISPR-Cas9-knockout experiments that a loss of furin does not prevent but substantially reduces S1/S2 cleavage at the SARS-CoV-2 spike, demonstrating that furin-mediated preactivation of the S protein in virus-infected cells is not necessary for triggering TMPRSS2-dependent cell–cell fusion. Moreover, they demonstrated that the mutation of the minimal cleavage site motif completely prevents syncytia formation. From these results, they concluded that proteolytic cleavage of the SARS-CoV-2 spike through furin is not essential but promotes both SARS-CoV-2 infectivity and cell–cell viral spread, while TMPRSS2 and ACE2 are essential for cell–cell viral spread. In another experiment, Follis et al. [21] demonstrated that the insertion of a furin recognition site at the S1/S2-junctional region of the SARS-CoV-1-spike glycoprotein enhances cell–cell fusion but does not affect virion entry. Menachery et al. [22] demonstrated that efficient cleavage of the MERS-CoV spike enables MERS-like coronaviruses from bats to infect human cells and inferred from their results that proteolytic cleavage of the spike protein is the primary barrier for zoonotic CoVs to human infection. Finally, Sanda et al. [23] detected using liquid chromatography − mass spectrometry the presence of O-glycans near the furin cleavage site and suggested that its cleavage is potentially regulated by the nearby O-glycans as described for other convertases. However, their presence and the possibility that glycan shielding plays an important role in the cleavage of SARS-CoV-2 spike are still under intense debate up to now [24].

In this study, we investigate the mechanism of proteolytic activation and conformational changes after proteolytic processing of the SARS-CoV-2 spike protein at the S1/S2 and S2′ cleavage sites, using molecular docking and MD simulation techniques. Moreover, we demonstrate the importance of the minimal cleavage site motif for enhancing furin binding and promoting TMPRSS2 proteolytic cleavage, preceding the disassembling process of the S protein. To conclude, we investigate the implication of glycans in the proteolytic mechanisms mediated by furin and TMPRSS2.

## Material and methods

### Model generation

**Fig. 1:**
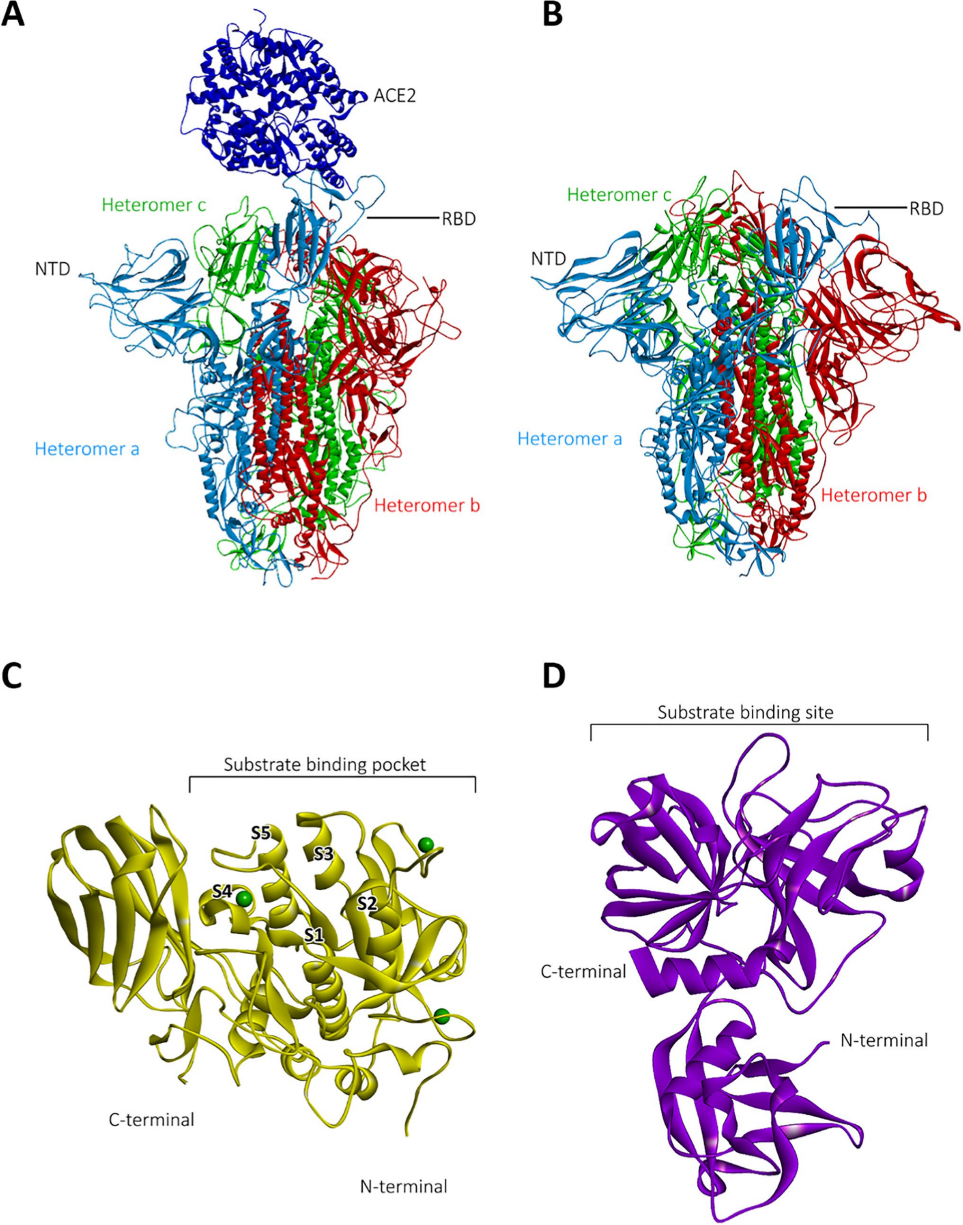
Tertiary structures obtained from homology modeling. S protein **A** with ACE2 (dark blue) and **B** without ACE2. S protein consists of three heteromers (light blue, red, green), each containing two subdomains S1 and S2. The S1 subunit (13–685 amino acids (aa)) is composed of the N-terminal domain (NTD) (13–303 aa) and RBD containing the C-terminal domain (CTD) (RBD: 319–541 aa with CTD: 334–527 aa), as well as the furin minimal cleavage site motif (682–685aa) at the boundary between the S1/S2 (685–886 aa). The S2 subunit (686–1273 aa) with the KR segment (814–815 aa) at S2′ site contains the virus fusion machinery. Note that the domains have been defined according to Ref. [39]. **C** Human furin and **D** human TMPRSS2

To generate the starting structures for the MD simulations of the S protein in the open-state conformation with ACE2 and in the closed-state conformation without ACE2, we performed 3D homology modeling using the SWISS-MODEL webserver (swissmodel.expasy.org) [25] in conjunction with the protein sequence of the S protein, derived in FASTA format from the sequence available in the NCBI database (ncbi.nlm.nih.gov) with accession number QIC53213.1 [26], and the template structures with pdb-codes 6ACG and 6ACC from the RCSB Protein Data Bank database (rcsb.org) [27, 28] for the ACE2-spike complex and spike without ACE2, respectively. We point out that the template structure 6ACG represents a SARS-CoV-spike glycoprotein binding with the cellular receptor ACE2 (ACE2-bound conformation 1), while the template structure 6ACC is a SARS-CoV-spike glycoprotein free of ACE2 with three RBDs in down conformation. Both structures have been determined by Song et al. [5] with cryogenic electron microscopy (cryo-EM) after proteolytic processing with trypsin and treatment at low pH. The model quality estimates for the homology models of the S protein without ACE2 and with ACE2 are GMQE = 0.74, QSQE = 0.93, and Seq. Identity = 76.83 and GMQE = 0.83, QSQE = 0.62, and Seq. Identity = 84.33, respectively. The homology modeled structures are visualized in Fig. 1A and B. We note that the amino acids 1–13 from the protein sequence were neglected by the SWISS-MODEL webserver and, thus, are not taken into account in these structures. To demonstrate the validity of our model generation procedure, we performed structural analysis and comparison of our ACE2-bound S-protein structure in the open-state conformation with respect to the structure published in the work of Amaro et al. [29] by using the Molecular Operating Environment (MOE) software [30]. For model building, the latter authors used the cryo-EM SARS-CoV-2 spike template structure with pdb-code 6VSB from the RCSB databank [31]. To further validate our modeled S1/S2 cleavage site, we compared it to the results of Raghuvamsi et al. [32], obtained from amide hydrogen–deuterium exchange mass spectrometry in conjunction with MD simulations. For a detailed description and discussion of the structural validation procedure, we refer to the section S2 in the supplement of our paper.

To perform a full N-/O-glycosylation of the S protein, we employed the Glycan Reader and Modeler tool implemented within CHARMM-GUI [33]. To this end, we docked a total of 66 N-glycans (22 N-glycans per monomer) and 4 O-glycans (2 O-glycans to chain A + 1 O-glycan to chain B + 1 O-glycan to chain C) to the S protein, as suggested in the studies of Refs. [34–36] and described in Tab. 1S-3S in the supplement of our paper. The resulting tertiary structure of the N-/O-glycosylated S protein is depicted in Fig. 6S of the supplement.

To generate the homology model of human furin with the SWISS-MODEL webserver, we used the FASTA sequence and template structure of the X-ray structure of human furin in complex with the competitive inhibitor meta-guanidinomethyl-Phac-RVR-Amba (pdb-code: 4OMC, GMQE = 0.97, Seq. Identity = 100). In case of TMPRSS2 it is worth mentioning that there is no experimental structure currently available [37]. We thus produced a homology model of this protease by employing the sequence from human TMPRSS2 (UniProtKB—O15393 (TMPS2_HUMAN)) and the crystal structure of the extracellular region of the transmembrane serine protease hepsin as a template (pdb-code: 1Z8G, GMQE = 0.48, Seq. Identity = 33.82), similar as in the work of Hussain et al. [38]. The resulting tertiary structures of furin and TMPRSS2 are shown in Fig. 1C and D.

All protein structures in this paper were generated with the software tool BIOVIA Discovery Studio Visualizer [40].

### Molecular docking

Protein–protein docking of furin and TMPRSS2 to the S protein and N-/O-glycosylated S protein was performed by using HEX 8.0.0 program [41]. The Critical Assessment of Predicted Interactions (CAPRI) protocol [42] was employed to predict the docking modes, and structures were docked for 1000 poses. The ranking was performed by estimating their free binding energy using Molecular Mechanics/Generalized Born Surface Area (MM/GBSA) approach [43]. The docking pose with the lowest binding energy was then used in subsequent MD simulations.

### MD simulations

In all our MD simulations, we used the GROningen MAchine for Chemical Simulations (GROMACS) program [44] in conjunction with the CHARMM36 forcefield. In addition, we employed full particle-mesh Ewald (PME) electrostatics with a Coulomb cutoff of 1.2 nm and computed the van der Waals (vdW) interactions using a vdW cutoff of 1.2 nm. We placed the protein complex into a cubic box and filled it with TIP3P water and added sodium ions to neutralize the system. The systems were prepared in an octahedron periodic box, using a minimum distance of 1 nm between the protein and the boundary. The resulting system sizes and atom numbers are given in Table 1.

**Table 1:** System sizes and atom numbers used in the calculations of this work

System	System size [nm^3^]	Number of atoms
S protein, closed state	45.287 × 45.287 × 45.287	8,502,338
Furin cleavage site-deleted S protein	53.640 × 53.640 × 53.640	12,423,872
S protein, open state + ACE2	51.875 × 51.875 × 51.875	10,678,485
S protein, open state + ACE2 + furin	53.665 × 53.665 × 53.665	12,424,114
S protein, open state + ACE2 + furin + TMPRSS2	64.796 × 64.796 × 64.796	15,733,515
N-/O-glycosylated S protein, open state + ACE2 + furin + TMPRSS2	64.841 × 64.841 × 64.841	15,735,681

Note that, to observe significant conformational rearrangement of the S protein, one needs to choose a solvation shell at least of the size selected in our paper, as will be shown in the following. First of all, we consider that the experiments of Ke et al. [45] based on cryo-electron microscopy and tomography have revealed that there is an average about one S trimer per 1,000 nm^2^ on the membrane surface of a SARS-CoV-2 virion and that S trimers are distributed randomly on the viral surface with no obvious clustering or interaction between them. Moreover, using cryo-electron tomography and cryo-subtomogram averaging, Tai et al. [46] have demonstrated that around 80% of the S proteins in the prefusion stage are at nearest neighbor distances ≥ 30 nm (see Fig. S3B in the supplementary information of their paper). From these works, we conclude that for all systems considered in our paper, the box size must be ≥ 30 nm, to enable significant conformational rearrangement to be captured.

To generate an isothermal–isobaric ensemble at the desired temperature and pressure, the system was equilibrated stepwise for 1 ns under isothermal–isochoric conditions, to adjust the temperature, and, then, for an additional 1 ns under isothermal–isobaric conditions, to adjust the temperature and pressure. We then performed production runs under isothermal–isobaric conditions for the timespans as described in the following. The thermostating and barostating of the system was carried out with the Bussi-Donadio-Parrinello thermostat with a time constant of *t*_*T*_ = 0.1 ps [47] and the Parrinello-Rahman barostat with a time constant of *t*_*P*_ = 2.0 ps, respectively [48]. For the numerical integration of the equations of motion, we used the leapfrog integrator with a timestep of 2 fs by applying H-bonds constraints. The initial MD simulations for the S-protein binding to ACE2 and without ACE2 binding were performed for 50-ns production run at the normal body temperature 310 K and a pressure of 1 bar (MD simulation phase A). Then, to the former case, we appended an additional 50-ns production run with an increased temperature at 314 K (high-fever temperature) but otherwise with the same conditions and parameters as previously (MD simulation phase B). We point out that the case without ACE2 binding at normal body temperature represents the case where no infection has occurred. By contrast, the case with ACE2 binding at high-fever temperature mimics the situation in patients with serious infection [49]. Such high fevers have, e.g., been found in clinical studies conducted on patients upon admission to the clinic, who died from COVID-19 [50]. To investigate the conformational changes of the uncleaved furin-S-protein complexes wild type with ACE2- (MD simulation phase C) and without ACE2-binding (MD simulation phase C1), furin cleavage site-deleted (mutated)-type (MD simulation phase C2), ACE2-TMPRSS2-furin-S-protein complex (MD simulation phase D) as well as furin- and TMPRSS2-cleaved ACE2-S-protein complex (MD simulation phase E), we performed additional 30-ns MD production runs at a temperature of 314 K and a pressure of 1 bar using the structures obtained from previous simulation phases as input. For more details, we refer to Tab. S4 in the supplement of our paper. Finally, we studied the influence of N-/O-glycosylation on the proteolytic process of the S protein mediated through furin and TMPRSS2 by carrying out a 300-ns MD simulation of the N-/O-glycosylated S protein in complex with furin and TMPRSS2 at a temperature of 314 K and pressure of 1 bar (MD simulation phase F).

## Results and discussion

### Furin and its minimum cleavage site

**Fig. 2:**
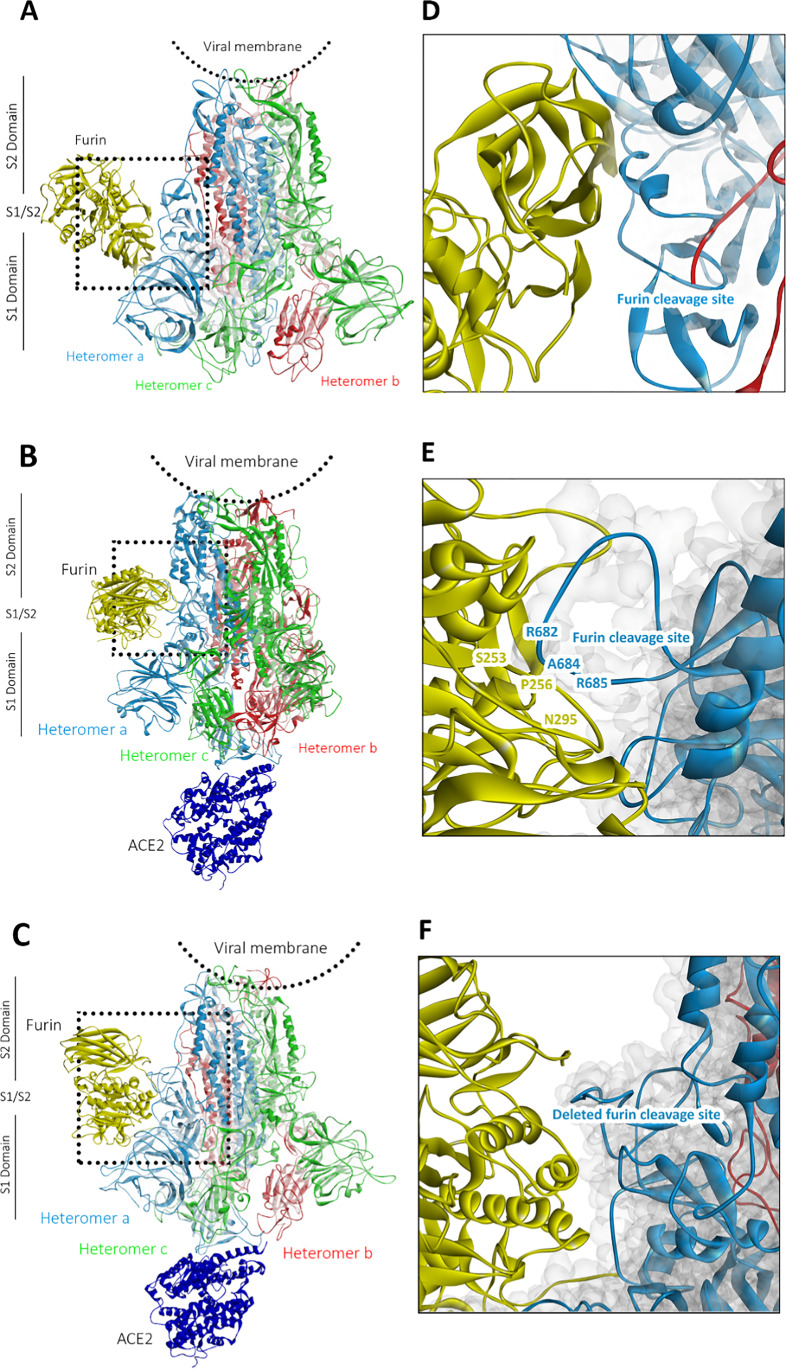
Final tertiary structures, obtained from our combined molecular docking and MD simulation procedure described in the “Material and methods” section of human furin (yellow) in complex with the S protein for the cases **A** without ACE2 (MD simulation phase C1) and **B** with ACE2 (MD simulation phase C) as well as **C** with the furin cleavage site-deletion variant of the S protein with ACE2 (MD simulation phase C2). The heterotrimers of the S protein are shown in red, green, and light blue colors, while the ACE2 is shown in dark blue color. **D**, **E,** and **F** are the corresponding close-up figures of the region near to the furin cleavage site or deleted furin cleavage site from the figures **A**, **B**, and **C**. **E** focuses on the amino acids, interacting at the interface between furin and its cleavage site. For more details about the MD simulation phases, we refer to the “MD simulations” section

To study the role of human furin and its minimum cleavage site, we consider in Fig. 2 the tertiary structures, obtained from our combined molecular docking and MD simulation procedure described in the “Material and methods” section, for the human furin in complex with the wild-type S protein with and without ACE2 as well as with the furin cleavage site-deletion variant of the S protein with ACE2.

**Fig. 3:**
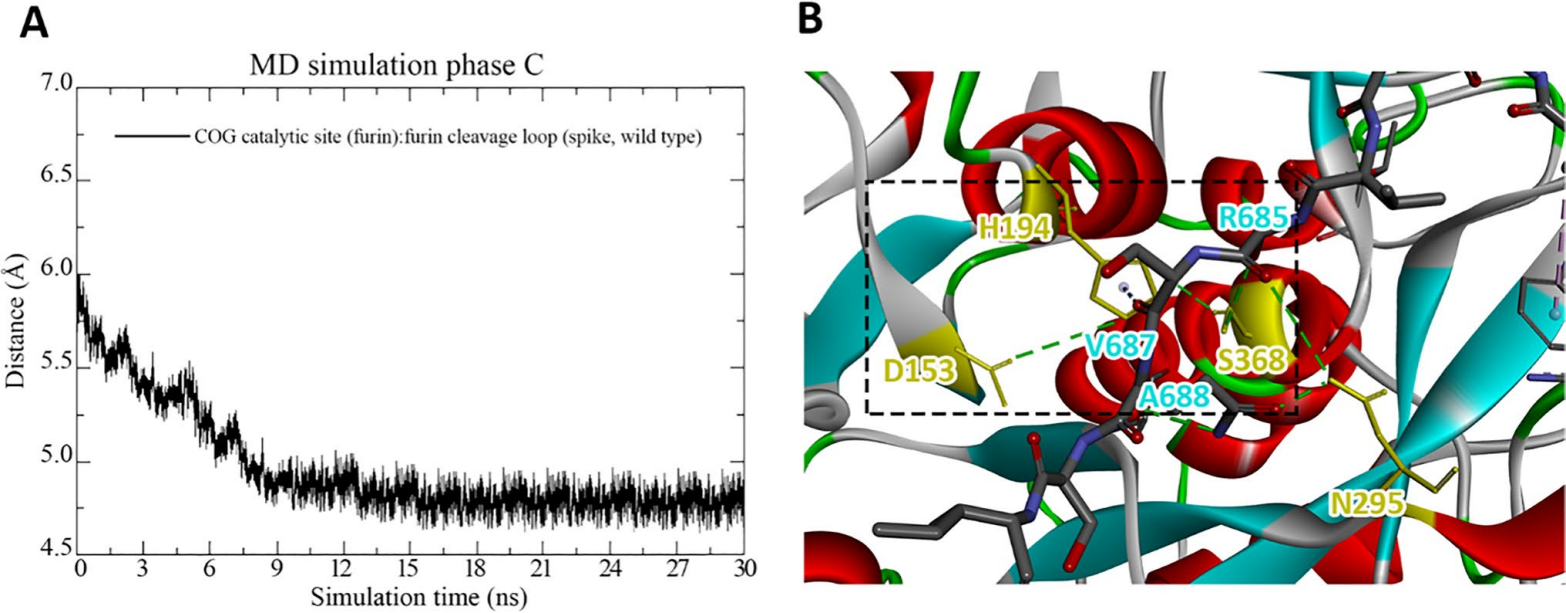
**A** Distance between the COGs of the catalytic site of furin, comprising Asp153, His194, and Ser368, and the furin cleavage loop on the wild-type S protein, consisting of the range of residues Ser680-Gln690, obtained from MD simulation phase C of the furin-ACE2-S-protein complex. **B** Close-up figure on the interactions between Asp153 and the other residues of the catalytic triad of furin (His194 and Ser368). Note that hydrogen bonds are shown in green color. For more details about the MD simulation phases, we refer to the “MD simulations” section

From the comparison of the structures of the first two cases in Fig. 2A and B, we deduce that furin binds more closely to the S protein, when ACE2 is bound to it, than in the case without ACE2 binding. This suggests that furin is attracted to the S protein in the former case. By further considering the corresponding close-up figures in Fig. 2D and E, we see that in the latter case, the minimal cleavage site is protruding out of the S protein and comes in closer contact with the furin than in the case without ACE2. This can also be concluded from Table 2, which shows that the S protein with ACE2 forms more molecular interactions with the furin protease compared to the case without ACE2 binding. This indicates that the binding of the ACE2 to the RBD of the S protein improves the accessibility of the furin to the minimal cleavage site of the corresponding heteromer, facilitating its proteolytic cleavage. To analyze if furin binds stably to the S protein enabling proteolytic processing, we consider next in Fig. 3A the distance between the center of geometries (COGs) of the catalytic site of furin, comprising the catalytic triad residues Asp153, His194 and Ser368 [51], and the furin cleavage loop on the wild-type S protein, consisting of the range of residues Ser680-Gln690, as a function of simulation time.

**Table 2:** Molecular interactions at the interface of the wild-type S protein in complex with furin for the cases with ACE2 (MD simulation phase C) and without ACE2 (MD simulation phase C1) as well as minimum cleavage site deleted S protein in complex with furin and ACE2 (MD simulation phase C2), obtained for the final tertiary structures visualized in Fig. 2. For more details about the MD simulation phases, we refer to the “MD simulations” section

Wild type								Mutate type			
With ACE2				Without ACE2				With ACE2			
Spike	Furin	Distance (Å)	Interaction type	Spike	Furin	Distance (Å)	Interaction type	Spike	Furin	Distance (Å)	Interaction type
ASP215	ARG130	1.73	H-Bond	GLN32	PRO458	4.56	Electrostatic	ARG233	ASP168	4.54	Electrostatic
ASP215	ARG130	1.66	H-Bond	ARG207	ASP460	4.25	Electrostatic	ARG233	ARG220	3.45	Electrostatic
GLN218	ASN325	2.30	H-Bond	HIS611	MET509	2.48	H-Bond	ASP234	TYR167	3.53	H-Bond
ASN606	GLY344	3.10	H-Bond	TYR622	GLU546	3.86	H-Bond	ALA307	LYS135	3.03	H-Bond
ARG682*	ASP228	5.02	Electrostatic	GLY625	PRO508	2.54	H-Bond	VAL308	ALA137	2.32	H-Bond
ARG682*	SER253	3.47	Electrostatic	ASN626	GLY510	2.69	H-Bond	ASP313	TYR142	3.31	H-Bond
ARG682*	TRP254	2.54	H-Bond	ASN627	THR413	2.6	H-Bond	ASN625	GLN439	3.45	H-Bond
ARG683*	ASP191	3.47	Electrostatic					ARG653	GLN111	3.15	H-Bond
ARG683*	ASP228	3.47	Electrostatic					TYR655	LYS117	3.67	H-Bond
ARG683*	ASP191	2.20	H-Bond					SER701	GLN399	3.55	H-Bond
ALA684*	PRO256	2.28	H-Bond					ALA707	LEU437	5.12	H-Bond
ARG685*	GLU236	5.23	Electrostatic					GLN709	GLY432	5.52	H-Bond
ARG685*	GLY255	2.01	H-Bond								
ARG685*	ASN295	2.54	H-Bond								
ARG685*	SER368**	3.48	H-Bond								
SER686	TRP254	2.54	H-Bond								
SER686	GLY255	2.04	H-Bond								
VAL687	HIS194**	5.36	Hydrophobic								
ALA688	ASN295	2.32	H-Bond								
SER940	TYR523	1.78	H-Bond								
SER940	TYR523	2.91	H-Bond								
ALA942	TYR523	3.77	Hydrophobic								

*4-minimal-residues-recognition motif for furin

**Catalytic triad of furin

We see that the curve decreases steadily up to a simulation time of 9 ns and afterwards reaches a plateau by oscillating around an average value of 4.7 Å up to the end of the simulation run, indicating that the catalytically relevant residues of furin bind stably directly to or nearby the furin cleavage loop of the wild-type S protein. A detailed analysis of the molecular interactions at the binding site of furin and the S protein in Table 2 reveals that in case of ACE2 binding to the S protein, all residues of the minimum cleavage site (Arg682, Arg683, Ala684, Arg685) and the residues of the catalytic triad of furin His194 and Ser368, which should directly bind to the cleavage site [8, 51, 52], are involved. Moreover, we deduce from Table 2 and Fig. 3B that Asp153 does not directly interact with the furin cleavage loop but forms a hydrogen bond between the carboxyl -O of Asp153 and the H atom of the NH group on the heterocyclic ring of His194 (black box). In addition, we see that another hydrogen bond is formed between the -OH of Ser368 and the second N atom of the same heterocyclic ring. We point out that, together with the interactions between the cleavage site and the residues His194 and Ser368 of the catalytic triad of furin mentioned previously, these hydrogen bond interactions are essential for proteolytic cleavage through furin [51, 52], indicating that this process can take place. Our result is in support of the studies of Hoffmann et al. and Papa et al., who demonstrated, respectively, that SARS-CoV-2 uses the receptor ACE2 for cell entry [18] and that furin cleavage promotes infection and cell–cell fusion [7]. Moreover, our study also reveals that furin binding is induced by ACE2 binding to the RBD of the S protein. This suggests that in cells with high furin level, the presence of furin nearby the S protein can lead to an acceleration of the proteolytic cleavage process, in concordance with the findings of Shang et al. [53].

To assess the importance of the 4-minimal-residues-recognition motif Arg682–Arg683-Ala684-Arg685↓ at the furin cleavage site for furin binding, we consider next the tertiary structure of the minimum cleavage site deleted S protein, where the 4-minimal-residues-recognition motif has been deleted, in complex with furin and ACE2. From Fig. 2C, we can deduce that the furin does not bind closely and specifically to the mutated site of the mutated S protein. This is confirmed by comparing in Table 2 the molecular interactions of the mutated- and wild-type cases with ACE2, showing that in the former case, less molecular interactions are present, and no binding to the mutated site does occur. Although the binding takes place in the same area as in case of the wild-type case as can be inferred from the close-up Fig. 2F, there are no interactions between the residues of the furin and the ones of the mutated S1/S2 region of the mutated S protein. This suggests that the 4-minimal-residues-recognition motif Arg682–Arg683-Ala684-Arg685↓ is important for furin-mediated proteolytic cleavage of the S protein. We point out in this regard that these amino acids agree with the minimal furin sequence Arg-X-X-Arg↓ [19, 54], necessary for furin binding and proteolytic cleavage, and are closely related to the furin consensus sequence Arg-X-[Lys/Arg]-Arg↓ [54, 55]. Note that a previous study [54] suggested based on biochemical experiments with two *bona fide* in vivo furin substrates—anthrax toxin protective antigen and avian influenza virus hemagglutinin—that the Arg residues at the positions P1 and P4 of a furin cleavage site of the type P4-P3-P2-P1↓ are essential for furin cleavage, while the amino acid (Arg/Lys) at position P2 is not essential but enhances the processing efficiency.

### ACE2 and furin preactivation in TMPRSS2 proteolytic cleavage process

**Fig. 4:**
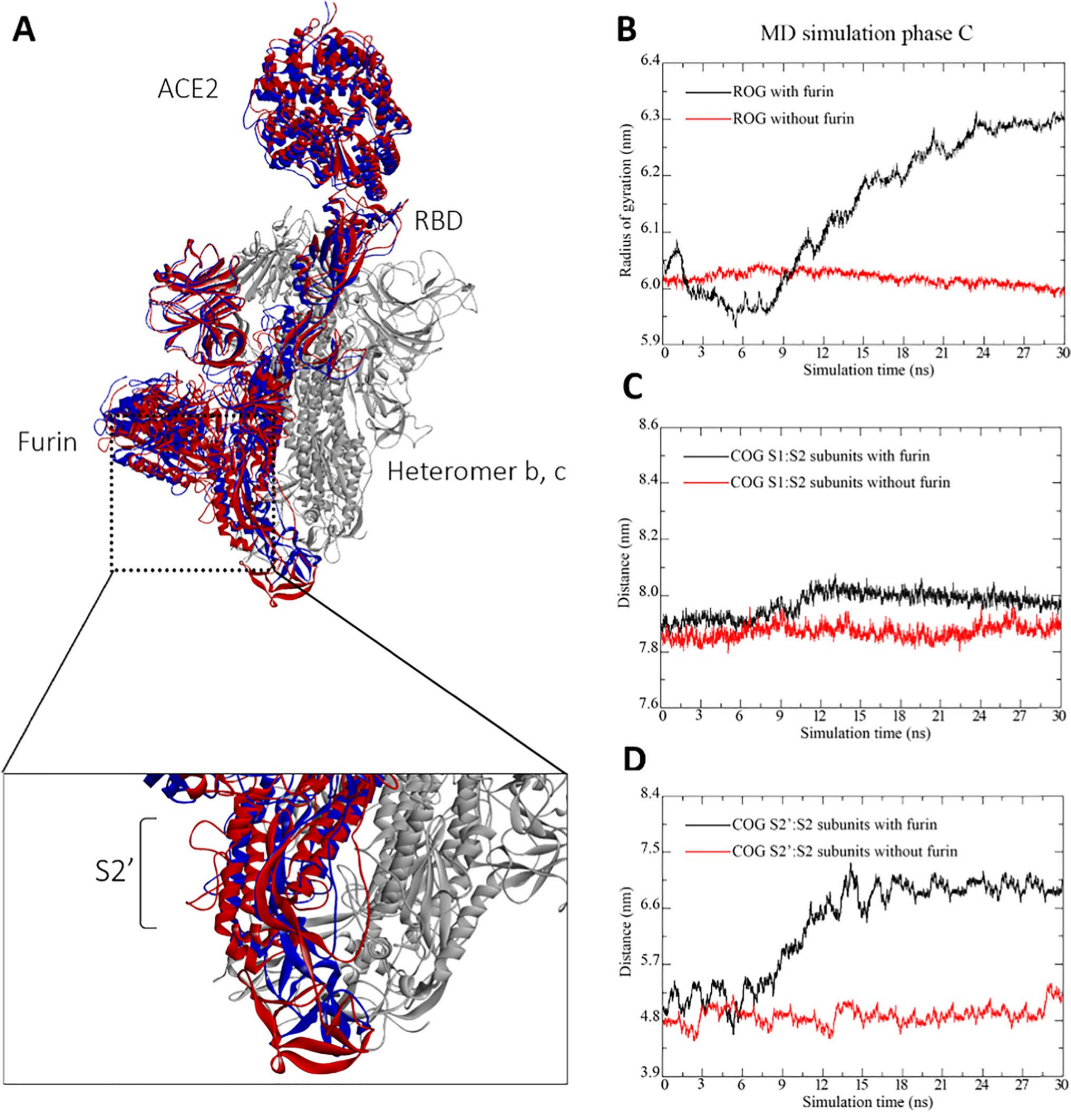
Results from MD simulation phase C of the furin-ACE2-S-protein complex. **A** Superposition of tertiary structures of the starting configuration (blue) and final configuration (red). **B** Radius of gyration (ROG) of the complex with furin binding (black curve) and without furin binding (red curve). **C** Distance between the COGs of the S1 and S2 subunits of the ACE2-binding heteromer of the S protein for the cases with furin binding (black curve) and without furin binding (red curve). **D** Distance between the COGs of the S2 and S2′ subunits of the ACE2-binding heteromer of the S protein for the cases with furin binding (black curve) and without furin binding (red curve). The subunits are defined as follows: S1, 13–685 aa; S2, 686–1273 aa; S2′, 800–820 aa. For more details about the MD simulation phases, we refer to the “MD simulations” section

To elucidate the role of the ACE2 and furin preactivation in the TMPRSS2 proteolytic cleavage process of the S protein, we compare next in Fig. 4 the results of the MD simulation of the ACE2-S-protein complex binding with furin to the case without furin binding, obtained from our combined molecular docking and MD simulation procedure described in the “Materials and methods” section.

From the comparison of the tertiary structures of the starting configuration and final configuration of the whole complex and the S2′ site in the close-up figure visualized in Fig. 4A, we can deduce that the S2′ site is projected out of the S protein, indicating that ACE2 and furin binding promotes TMPRSS2 binding. Since in a recent experimental work of Papa et al. [7], it has been found that TMPRSS2 proteolytic cleavage initiated by ACE2 binding is essential for viral infection and cell–cell viral spread (but not furin binding), and it is worth analyzing in the following effect of furin on this process. In Fig. 4B, we show the curves of the ROGs for the cases with furin binding and without furin binding to the ACE2-S-protein complex as a function of simulation time. It is worth mentioning in this regard that the ROG is an indicator of protein structure compactness [56]. We see that, while the curve of the complex without furin remains nearly close to a value of 6.0 nm throughout the whole simulation run, the ROG curve for the case with furin binding is subjected to a significant increase after a simulation time of 7.5 ns and stabilize at the end of the simulation run at a value of 6.3 nm, indicating a less tight packing of the protein complex than in the former case. In the following, we analyze in more detail the subunits, contributing to the decrease of compactness of the complex after furin binding. To this end, we consider, respectively, in Fig. 4C and D the distances between the COGs of the S1 and S2 subunits as well as of the S2 and S2′ subunits of the ACE2-binding heteromer of the S protein for the cases with furin binding (black curve) and without furin binding (red curve). By comparing these results to the ones in Fig. 4B, we conclude that the increase of the ROG curve at 7.5 ns for the case with furin binding correlates with a moderate increase of the distance between the COGs of the S1 and S2 subunits as well as a large increase of the distance between the COGs of the S2 and S2′ subunits. By contrast, without furin binding, the ROG and these distances remain stable throughout the simulation run, similarly as observed in case of the ACE2-S-protein complex at body temperature (see Fig. 1S in the supplement of our paper). From these observations, we conclude that furin binding promotes the separation of the S1 and S2 subunits and supports TMPRSS2 binding through protruding of the S2′ subunit out of the S protein. Our results are in support of the findings of Papa et al. [7], who found that furin cleavage of the S protein promotes but is not essential for viral infection and cell–cell viral spread.

### Preactivation of the S protein through TMPRSS2

**Fig. 5:**
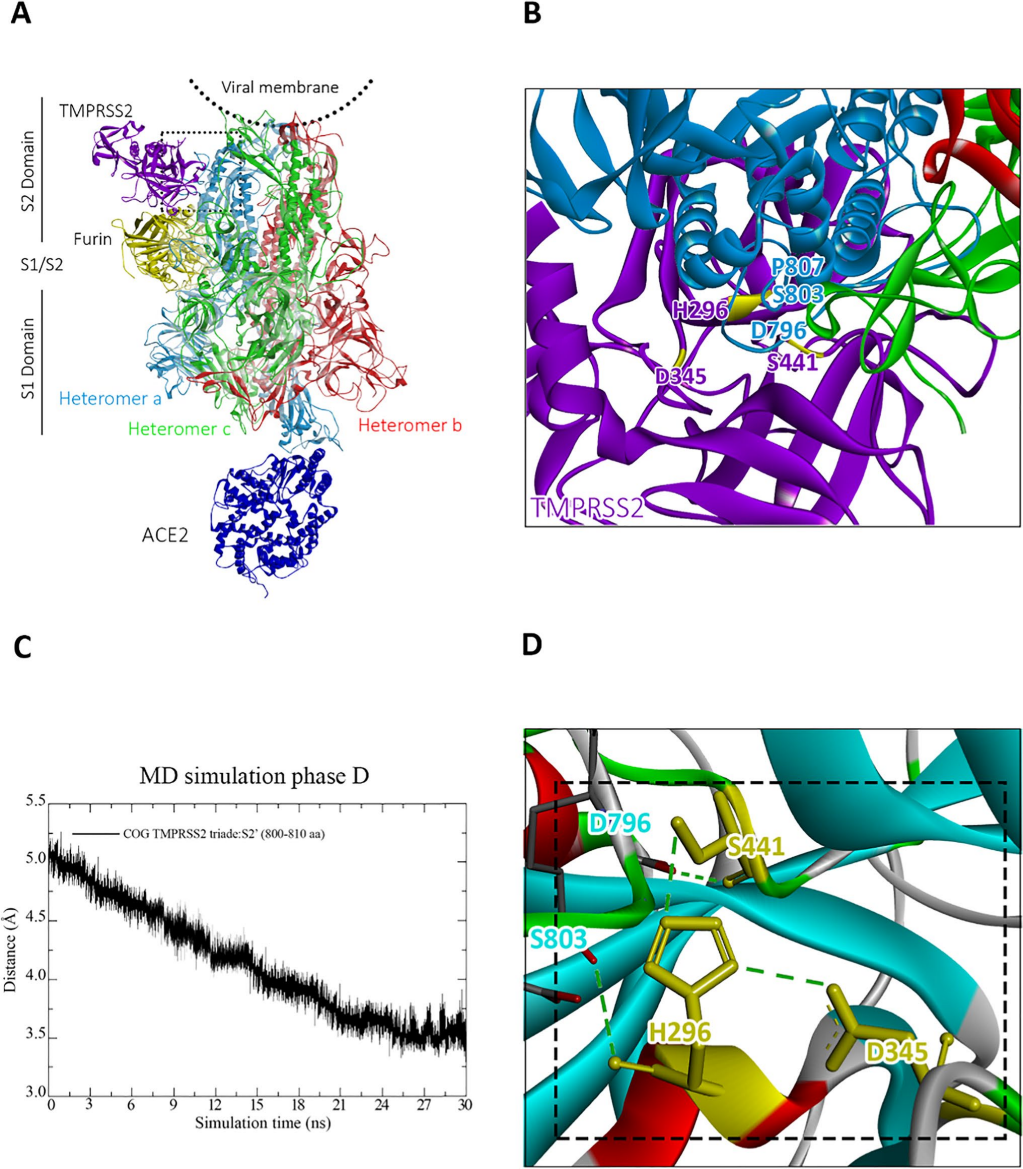
Results obtained from MD simulation phase D of the TMPRSS2-furin-S-protein complex. **A** Final tertiary structure of the whole complex. The heterotrimers of the S protein are shown in red, green, and light blue colors, while ACE2, furin, and TMPRSS2 are shown in dark blue, yellow, and purple colors, respectively. **B** Close-up figure of the amino acids of the catalytic triad of TMPRSS2 at the S2′ site of the S protein. Yellow segment part shows the catalytic triad of TMPRSS2 (His296, Asp345, Ser441). **C** Distance of the COGs of the amino acids of the catalytic triad of TMPRSS2 and S2′ site of the S protein (S2′: 800–820 aa). **D** Close-up figure on the interactions between Asp345 and the other residues of the catalytic triad of TMPRSS2 (His296 and Ser441). Note that hydrogen bonds are shown in green color. For more details about the MD simulation phases, we refer to the “MD simulations” section

To study the preactivation process of the S protein prior to TMPRSS2 proteolytic cleavage, we consider next the results obtained from MD simulation of the TMPRSS2-furin-S-protein complex in Fig. 5.

From Figs. 5A and B, we infer that the catalytic site of TMPRSS2 binds to the S2′ site of the S protein, despite furin binding to the S protein. To investigate, if the TMPRSS2 proteolytic cleavage after furin preactivation can take place, we analyze in the following the TMPRSS2-binding process in more detail. In Fig. 5C, we show the distance of the COG of the amino acids of the catalytic triad of TMPRSS2 (His296, Asp345, Ser441) and S2′ site of the S protein (range of amino acids 800–810).

We see that the curve decreases steadily up to a simulation time of 25 ns and then reaches a plateau up to the end of the simulation run by fluctuating about an average value of 3.6 Å. From this result, we conclude that the catalytic site of TMPRSS2 is attracted and stably bound to the S2′ site of the S protein. In Table 3, we show the molecular interactions formed between both sites in the final configuration, obtained from MD simulation.

**Table 3:** Molecular interactions between TMPRSS2 and S protein in complex with ACE2 and furin at the end of MD simulation D. For more details about the MD simulation phases, we refer to the “MD simulations” section

Spike	TMPRSS2	Distance (Å)	Interaction type
ILE794	GLU389	3.44	H-Bond
ASP796*	SER441**	2.32	H-Bond
SER803*	GLN276	2.94	H-Bond
SER803*	HIS296**	2.82	H-Bond
PRO807*	GLN276	3.28	H-Bond
SER810*	GLY391	2.56	H-Bond
ASP936	GLN317	2.98	H-Bond

*TMPRSS2 cleavage site S2′

**Catalytic triad of TMPRSS2

We see that TMPRSS2 forms hydrogen-bond interactions with the S2′ site of the S protein, involving His296 and Ser441 from its catalytic triad. From previous works [57, 58], it is well-known that these amino acids are required for proteolytic cleavage of the S protein through TMPRSS2, in addition to Asp345. Moreover, we notice that Asp345 does not directly interact with the S2′ site (similarly as Asp153 of furin, which does not directly interact with the furin cleavage loop on the S protein (see the “Furin and its minimum cleavage site” section)). To analyze this aspect in more detail, we show in Fig. 5D a close-up figure on the interactions between Asp345 and the other residues of the catalytic triad of TMPRSS2. We see that Asp345 forms a hydrogen bond between the carboxyl -O of Asp345 and the H atom of the NH group on the heterocyclic ring of His296 as well as another hydrogen bond between the -OH of Ser441 and the second N-atom of the same heterocyclic ring (black box). We note that, together with the interactions between the S2′ site and the residues His296 and Ser441 of the catalytic triad of TMPRSS2 discussed previously, these hydrogen bond interactions are essential for proteolytic cleavage [52]. This indicates that, similarly as the furin proteolytic cleavage process discussed previously, also the TMPRSS2-mediated proteolytic process can take place.

### Proteolytic cleavage of the S protein through furin and TMPRSS2

**Fig. 6:**
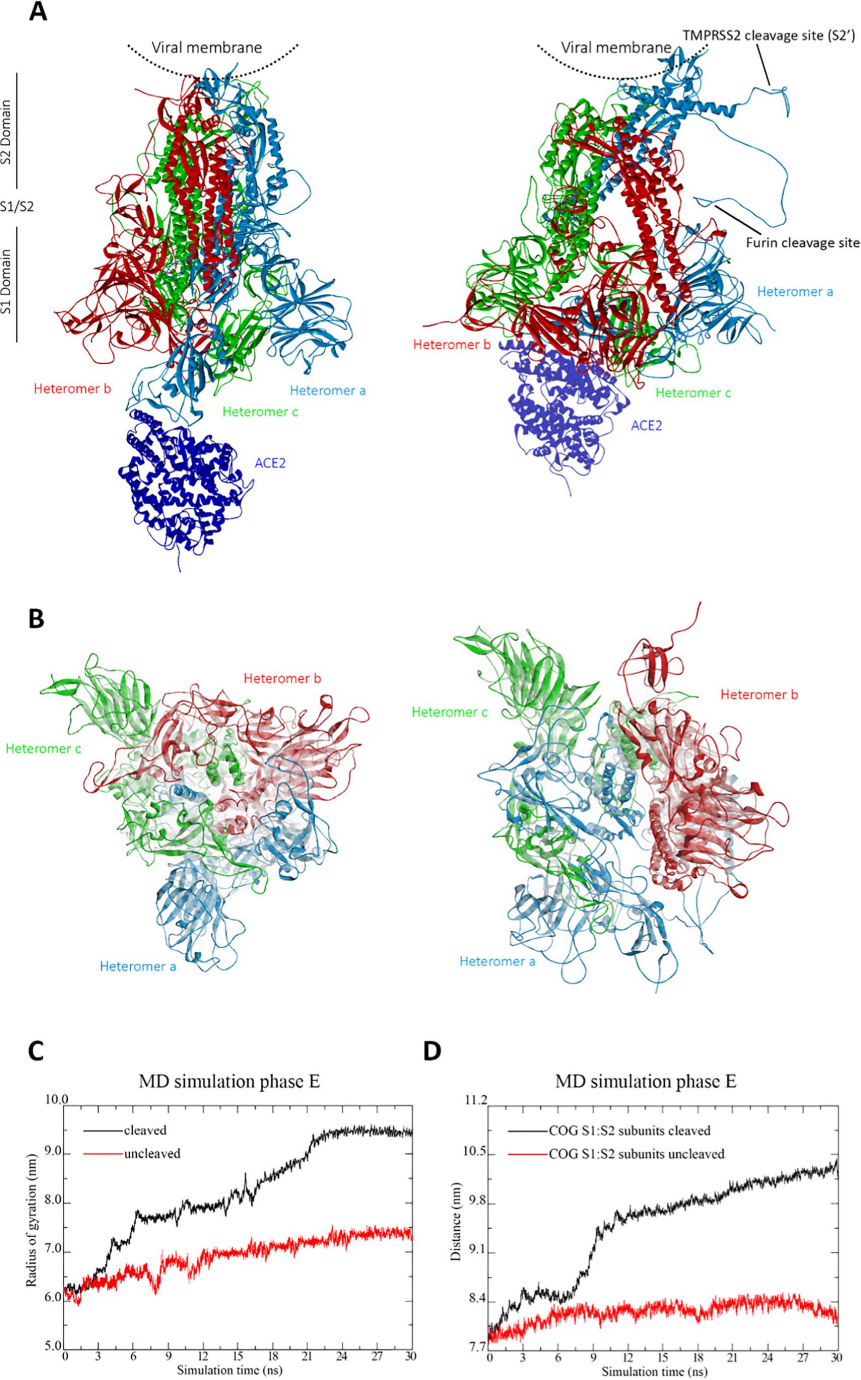
Tertiary structures of the S protein in complex with ACE2 before (left) and after furin and TMPRSS2 cleavage (right) from the end of MD simulation phase E. **A** Side view. **B** Top view. The heteromers of the S protein are shown in red, green, and light blue colors, while ACE2 is shown in dark blue color. **C** Radius of gyration of the S protein and **D** distance of the COGs of the S1 and S2 subunits after proteolytic cleavage (black curve) from MD simulation phase E in comparison to the uncleaved case (red curve) from MD simulation phase D. The subunits are defined as follows: S1, 13–685 aa; S2, 686–1273 aa. For more details about the MD simulation phases, we refer to the “MD simulations” section

To investigate the effect of proteolytic cleavage on the S protein, we consider next the MD simulation results obtained after cleavage of the S protein at the S1/S2 and S2′ sites through furin and TMPRSS2, respectively. From the comparison of the tertiary structures in side view perspective of the S protein in complex with ACE2 before (left) and after furin and TMPRSS2 cleavage (right) in Fig. 6A, we conclude that proteolytic cleavage at the S1/S2 and S2′ sites leads to a separation of the subunits of the cleaved heteromer and to a protruding of the furin and TMPRSS2 cleavage sites out of the S protein. Overall this results in a disassembling of the S protein, as can be further concluded from Fig. 6B and C, showing, respectively, the S protein before (left) and after proteolytic cleavage (right) from a top view perspective and the ROG of the cleaved S protein in comparison to the uncleaved case.

The increase of the ROG in Fig. 6C goes along with a separation of the subunits S1 and S2, as can be inferred from the increase of the distance of the COGs of the S1 and S2 subunits after proteolytic cleavage (black curve) in comparison to the uncleaved case (red curve) in Fig. 6D. These results demonstrate that the expansion of the S protein after furin- and TMPRSS2 cleavage is accompanied by a substantial conformational change. A more detailed analysis of the tertiary structure of the cleaved complex in Fig. 6A reveals that the S1 and S2 subunits are subjected to significant structural changes. The S1 subunit reorients, whereas parts of the S2 subunit dissociates from the complex. These observations agree well with several experimental studies on SARS-CoV-2 and its predecessors. For instance, several experimental investigations on former coronaviruses, reviewed in references [59, 60], and [61], indicated that the cleavage at the S1/S2 site may be crucial for conformational changes required for receptor binding and/or subsequent exposure of the S2′ site to host proteases during viral entry [15]. Bestle et al. [15] suggested that SARS-CoV-2 uses the furin and TMPRSS2 proteases to cleave the region between the S1 and S2 subunits and that afterwards it uses the S2 subunit as the membrane fusion part, to fuse it with the host membrane and transfer its RNA in an additional step. Wrobel et al. [62] determined by comparing cryo-EM structures of the spike of RaTG13, known as the closest relative of SARS-CoV-2, but lacking the furin cleavage site at the S1/S2 junction, with the furin-cleaved- and uncleaved-SARS-CoV-2 spike protein that cleavage at the furin cleavage site, decreases the overall stability of SARS-CoV-2 spike protein and facilitates the adoption of the open conformation that is required for the spike protein to bind to the ACE2 receptor. Shang et al. [53, 63] inferred from a further experimental study by comparing the binding affinities of ACE2 to SARS-CoV-2 RBD and SARS-CoV-1 RBD that SARS-CoV-2 RBD, albeit more potent, is less exposed than SARS-CoV-1 RBD. They suggested that the hidden RBD could allow SARS-CoV-2 to evade immune surveillance while maintaining efficient cell entry through furin preactivation, which may contribute to the wide spread of the virus. Furthermore, the recent SARS-CoV-2 variants B.1.1.7 [64], B.1.617.2 [65], and B.1.1.529 [66], possessing the mutations at the RBD and amino acid position 681 (B.1.1.7 and B.1.1.529: P681H [65, 67], B.1.617.2: P681R [66]) adjacent to the furin cleavage site leading, respectively, to increased binding affinities of RBD to ACE2 and of furin to the furin cleavage site, were found to be more transmissible and to cause more severe illness than pre-existing SARS-CoV-2 variants. This suggests that the ACE-mediated furin cleavage process, even if not essential for viral infection and cell–cell viral spread, is a major catalyst of the disease [68].

### Influence of glycans on the proteolytic process of the S protein through furin and TMPRSS2

**Fig. 7:**
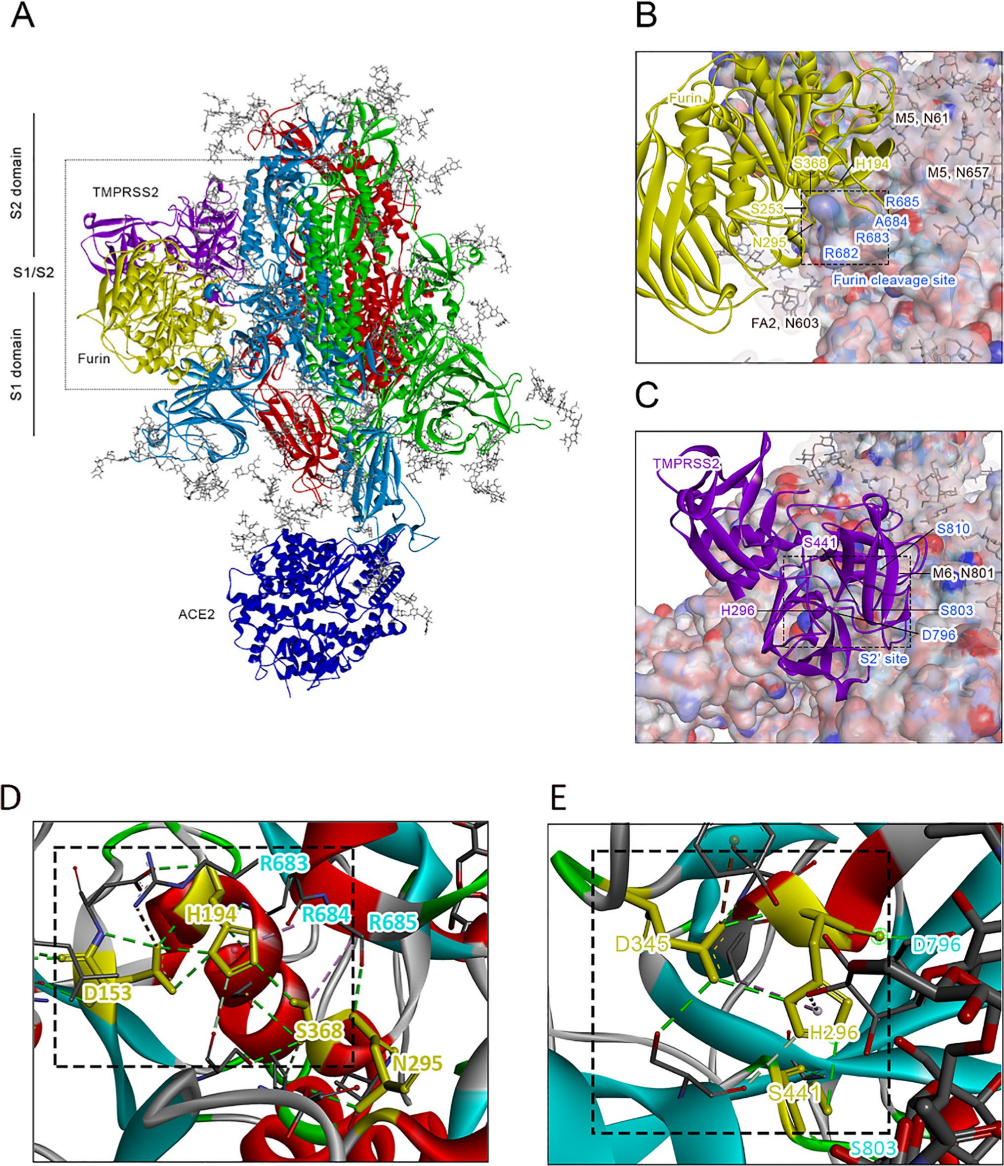
Tertiary structures of the ACE2-bound N-/O-glycosylated S protein near the cleavage sites of furin as well as TMPRSS2 after 300-ns MD simulation (MD simulation phase E). **A** Main part of the ACE2-bound N-/O-glycosylated S protein in complex with furin as well as TMPRSS2. **B** Close-up figure of ACE2-bound N-/O-glycosylated S protein near the furin cleavage site. **C** Close-up figure of ACE2-bound N-/O-glycosylated S protein near the TMPRSS2 cleavage site. The heteromers of the S protein are shown in red, green, and light blue colors, while ACE2, furin, and TMPRSS2 are shown in dark blue, yellow, and purple colors, respectively. **D** Close-up figure on the interactions between Asp153 and the other residues of the catalytic triad of furin (His194 and Ser368). **E** Close-up figure on the interactions between Asp345 and the other residues of the catalytic triad of TMPRSS2 (His296 and Ser441). Note that hydrogen bonds are shown in green color. For more details about the MD simulation phases, we refer to the “MD simulations” section

To investigate the influence of N-/O-glycosylation on the proteolytic process of the S protein through furin and TMPRSS2, we consider next in Fig. 7A–C the tertiary structure of the ACE2-bound N-/O-glycosylated S protein at the cleavage sites of furin as well as TMPRSS2 after 300-ns MD simulation. From Fig. 7B, we deduce that in case of furin, there are two M5 glycans at the N-glycosylation sites N61 and N657, which are located nearby the furin cleavage site of the S protein. To check how severely these glycans affect the binding of furin to the S protein and related proteolytic process, we consider next in Table 4 (left) the results for the molecular interactions between the ACE2-bound N-/O-glycosylated S protein and furin, obtained after 300-ns MD simulation. We notice that, similar to the case without glycans, the residues of the catalytic triad of furin His194 and Ser368 directly bind to the cleavage site of the ACE2-bound N-/O-glycosylated S protein. Moreover, we deduce from Fig. 7D that Asp 153 forms a hydrogen bond between the carboxyl -O of Asp153 and the H atom of the NH group on the heterocyclic ring of His194 as well as another hydrogen bond between the -OH of Ser368 and the second N atom of the same heterocyclic ring (see black box in both cases). This demonstrates that our results, obtained in the case without glycans are reproducible in the case with glycans and that glycans do not prevent the proteolytic process mediated by furin. In case of the TMPRSS2 cleavage site, we conclude from Fig. 7C that there is only one glycan of the M6 type located nearby the S2′ region of the S protein. We note that the M6 glycan binds to the S protein via N-glycosylation site at N801, which is located directly at the interface between TMPRSS2 and S2′ site and, thus, might influence the proteolytic process. To analyze this aspect in more detail, we consider in Table 4 (right) the results for the molecular interactions between the ACE2-bound N-/O-glycosylated S protein and TMPRSS2, obtained after 300-ns MD simulation. We see that similar to the case without glycans, TMPRSS2 forms hydrogen bond interactions with the S2′ site of the S protein, involving His296 and Ser441 from its catalytic triad. Moreover, we infer from Fig. 7E that a hydrogen bond between the carboxyl -O of Asp345 and the H atom of the NH group on the heterocyclic ring of His296 as well as another hydrogen bond between the -OH of Ser441 and the second N atom of the same heterocyclic ring is formed (see black box). From these observations, we conclude that similarly to furin, our TMPRSS2 results obtained in the case without glycans are reproducible in the case with glycans, which demonstrates that glycans also do not prevent the proteolytic process mediated by TMPRSS2.

**Table 4:** Molecular interactions between furin and S protein (left) as well as TMPRSS2 and S protein (right) in complex with ACE2 and O- as well as N-glycans from the end of MD simulation phase E. For more details about the MD simulation phases, we refer to the “MD simulations” section

Spike	Furin	Distance (Å)	Interaction type	Spike	TMPRSS2	Distance (Å)	Interaction type
ARG214	ALA404	4.82	Hydrophobic	ASP796***	His296****	3.26	H-Bond
ARG214	ASP430	2.97	H-Bond	SER803***	SER441****	3.89	H-Bond
ARG214	GLN129	2.08	H-Bond	SER803***	GLY439	2.72	H-Bond
ASP215	HIS405	2.46	H-Bond	SER810***	TYR414	4.68	H-Bond
ASP215	HIS405	5.15	Electrostatic	LEU841	ARG470	4.23	H-Bond
PRO217	HIS405	4.83	Hydrophobic				
GLN218	PHE323	2.08	H-Bond				
GLN218	HIS405	2.97	H-Bond				
ASN606	ASN347	2.54	H-Bond				
SER680	MET189	3.29	H-Bond				
SER680	ASN190	2.16	H-Bond				
ARG682*	SER253	2.63	H-Bond				
ARG683*	ASP191	2.16	H-Bond				
ARG683*	ASP153**	4.26	Electrostatic				
ARG683*	ASP154	5.06	Electrostatic				
ALA684*	HIS194**	5.36	Hydrophobic				
ALA684*	HIS364	4.92	Hydrophobic				
ALA684*	ASN295	2.50	H-Bond				
ALA684*	GLY366	3.60	H-Bond				
ARG685*	TRP328	5.49	Hydrophobic				
ARG685*	SER368**	5.21	Hydrophobic				
ARG685*	TYR329	5.17	Hydrophobic				
VAL687	TRP328	4.14	Hydrophobic				
VAL687	TRP328	3.37	Hydrophobic				
SER689	GLN350	2.61	H-Bond				

*4-minimal-residues-recognition motif for furin

**Catalytic triad of furin

***TMPRSS2 cleavage site S2′

****Catalytic triad of TMPRSS2

To conclude, it is worth pointing out that furin is highly expressed in the cells of the respiratory tract, which renders it more likely that SARS-CoV-2 exploits this convertase to activate its surface glycoprotein [16, 69, 70] and increases its infectivity [71]. Moreover, aberrant expression or activity of furin has been suspected to lead to a variety of disorders, e.g., cancer, diabetes, inflammation, neurological diseases, and cardiovascular diseases [9]. For example, several functional studies confirmed the expression of furin in a large variety of cancers such as head and neck squamous cell carcinoma, breast cancer, and rhabdomyosarcoma and strongly indicate that high furin activity promotes the malignant phenotype of cancer cells [72]. Moreover, Wang et al. [73] found by analyzing blood plasma obtained from acute myocardial infarction patients, which were predominantly male (63%) with a median age of 66 years, that elevated furin levels are associated with all-cause mortality and recurrent cardiovascular events [73]. Another study from Fernandez et al. revealed that a high furin concentration in the plasma is associated with an elevated risk of diabetes mellitus and premature mortality [74]. From these works, we deduce that a high furin levels are primarily encountered in diseases affecting predominantly older male patients and goes along with an increased severity of the disease. Considering that the furin cleavage process is an important step of viral entry as well as cell–cell viral spread of SARS-CoV-2 in in vivo systems (additional evidence is provided in section S5 in the supplement of our paper), we conclude that previously described risk factors, correlating with a high furin level, are likely to increase the virulence of the viral infection by SARS-CoV-2. In contrast to that, from SARS-epidemiologic data and their study with SARS-CoV-1-infected rats, Xie et al. [75] inferred that the more elevated ACE2 level is responsible for the obvious predominance of young adult patients with a slight female proneness in SARS attacks. All these investigations indicate that the high furin level (and not the high ACE2 level) is responsible for the age- and gender-dependence of mortality of COVID-19 patients, affected by the risk factors previously mentioned. A high level of furin increases the chance for a successful proteolytic cleavage of the S protein, which increases the probability for viral infection and an efficient cell–cell viral spread. Finally, it is also worth mentioning that, besides corona viruses, furin‐mediated cleavage has also been discovered in a wide range of evolutionary diverse virus families, including herpes, HIV, influenza, dengue, Ebola, and Marburg viruses [9], which shows that its optimization might lead to an evolutionary advantage for the new virus variants.

## Summary and conclusions

We conclude from our large-scale MD simulations that the open state of SARS-CoV-2 spike protein shows a very stable complex with the host cell receptor ACE2 both at body and fever temperatures. A detailed analysis of the complex reveals that the furin cleavage site of the ACE2-preactivated SARS-CoV-2 spike protein protrudes out of the spike. This result suggests that ACE2 binding promotes the release of the furin cleavage site from the spike protein and facilitates furin binding, enabling proteolytic cleavage of the spike protein and viral entry in a further step. From the subsequent molecular docking of furin to ACE2-preactivated SARS-CoV-2 spike protein and a large-scale MD simulation, we deduce that the two amino acids of the catalytic triad of furin, His194, and Ser368 directly bind to the SARS-CoV-2 spike protein at the furin cleavage site while forming a hydrogen bond to each other, and the third one, Asp153, forms a hydrogen bond with His194. We infer from these results that proteolytic cleavage mediated by furin can take place at the high fever temperature under investigation. By contrast, the results in the case without ACE2 binding concord with the furin cleavage site-deleted SARS-CoV-2 spike case, showing that ACE2 binding is a prerequisite for the furin cleavage process. Furthermore, the MD simulation results of the ACE2-furin-SARS-CoV-2 spike complex reveal that furin binding promotes the separation of the S1 and S2 subunits and supports TMPRSS2 binding through protruding of the S2′ subunit out of the spike. After molecular docking of TMPRSS2 to the latter complex and subsequent large-scale MD simulation, we find especially that TMPRSS2 forms molecular interactions with the S2′ site of the spike protein, involving His296 and Ser441 from its catalytic triad. Similar as in the furin case, the His and Ser form a hydrogen bond to each other, and a third amino acid from the triad, Asp345, forms a hydrogen bond to His296. We conclude from these results that proteolytic cleavage of the spike protein at the S2′ site through TMPRSS2 is enabled under the investigated conditions. Moreover, the MD simulation of SARS-CoV-2-spike protein after furin and TMPRSS2 cleavage shows that parts of the S2 subunit of SARS-CoV-2 spike protein dissociate from the complex, suggesting that the S2 subunit is used for fusion with the host membrane before transfer of the viral RNA into the host cell. Finally, repeating our calculations with the N-/O-glycosylated SARS-CoV-2 spike protein demonstrate that our results are reproducible and that glycans do not prevent the proteolytic processes mediated by furin and TMPRSS2.

## Supplementary Information

Below is the link to the electronic supplementary material.

Supplementary file1 (DOCX 4.10 MB)

## Data Availability

The datasets generated during and/or analyzed during the current study are available from the corresponding author on reasonable request.
