# Supplementary material for: Study of protease-mediated processes initiating viral infection and cell–cell viral spreading of SARS-CoV-2
Source: J Mol Model. 2022 Jul 19;28(8):224. doi: 10.1007/s00894-022-05206-8 (PMC9296015; doi:10.1007/s00894-022-05206-8)
Supplement: Supplementary file 1 — Supplementary file1 (DOCX 4.10 MB) [file 894_2022_5206_MOESM1_ESM.docx]

Supplementary Information for

**Study of protease-mediated processes initiating viral infection and cell-cell-viral spreading of SARS-CoV-2**

Thanawat Thaingtamtanha ^1^, Stephan A. Baeurle *^1^

1 Department of Chemistry and Biology, Universität Siegen, Adolf-Reichwein-Str. 2, D-57076 Siegen, Germany.

* Corresponding author: Stephan A. Baeurle

**Email:** Stephan.Baeurle@uni-siegen.de

**S1. MD simulations of the ACE2-S-protein complex (open state) and S protein (closed state)**


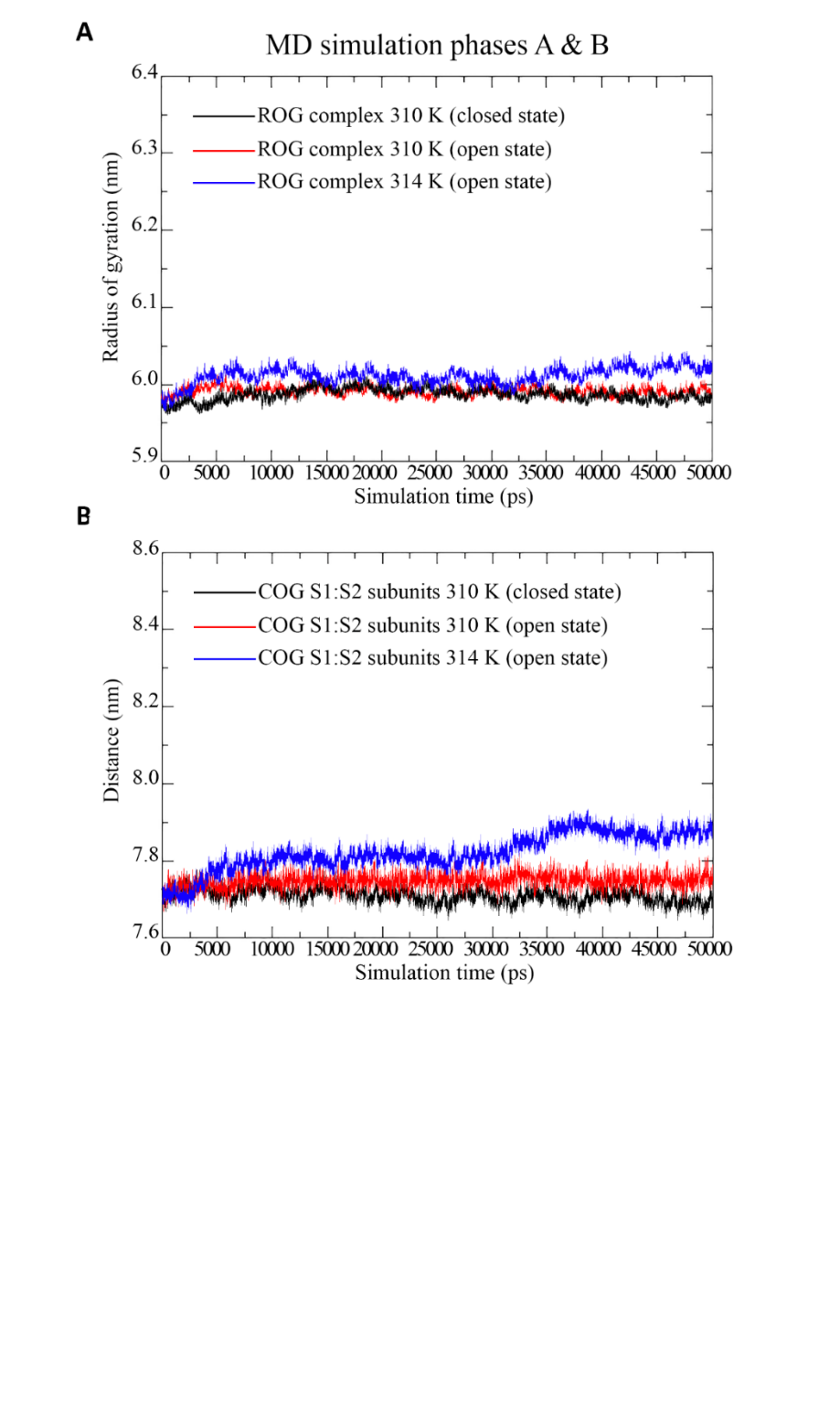


Fig. 1S: Results of the ACE2-S-protein complex (open state) and S protein (closed state) at different temperatures from MD-simulation phases A and B. (A) Radius of gyration (ROG). (B) Distance between the center of geometries (COGs) of the S1- and S2-subunits of the ACE2-binding heteromer of the S protein. The subunits are defined as follows: S1: 13-685 aa, S2: 686-1273 aa. For more details about the MD-simulation phases, we refer to section A.3.

**S2. Validation of the structural generation procedure**


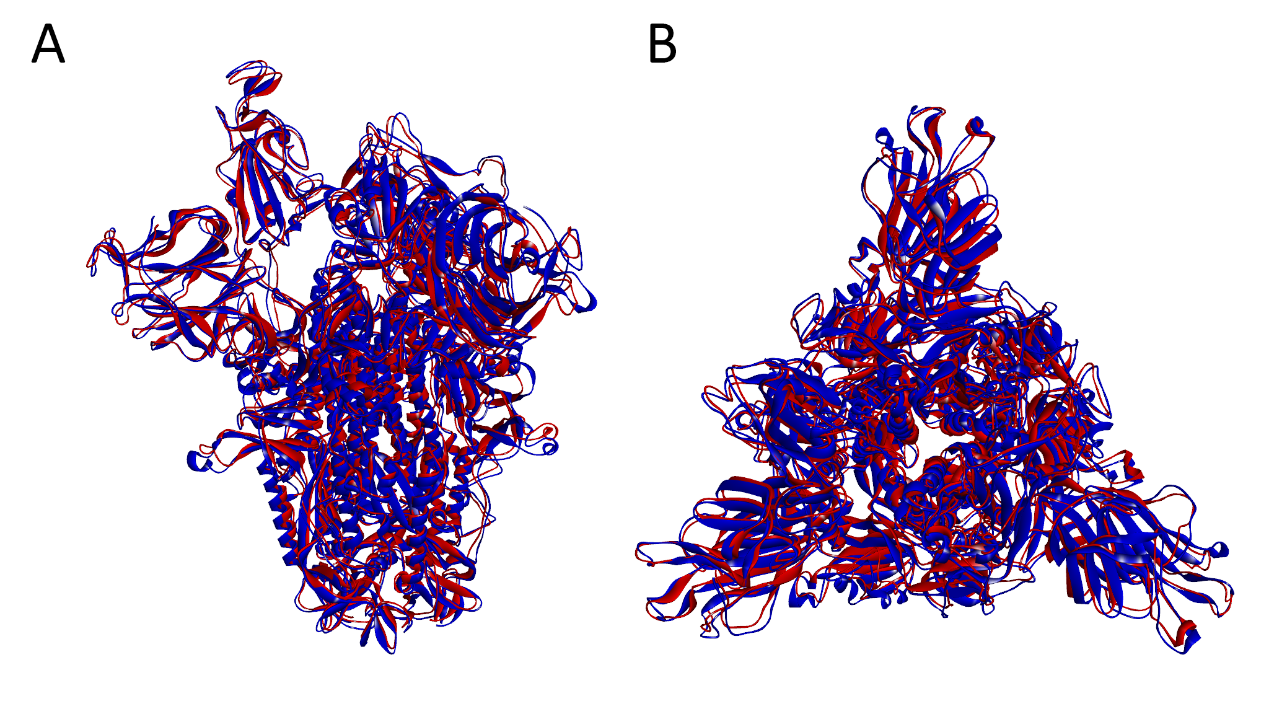


Fig. 2S: Comparison of tertiary structures of the S protein in the open state used as starting structures for the MD simulations of the ACE2-bound S protein (A) in our work and (B) in the work of Amaro *et al.* [red: our structure; blue: Amaro’s structure].

To validate our starting tertiary structure of the ACE2-bound S protein used in our MD simulations in the case without glycans, we performed structural analysis and comparison of our structure with respect to the structure published in the work of Amaro *et al.* by using the Molecular Operating Environment (MOE) software [1]. In their study Amaro *et al.* [2] constructed their starting model in several steps with homology modeling using Modeller v9.19 [3,4] and with i-TASSER [5-7], which relies on fold recognition (or threading) for template detection and reassembling structural fragments from threading templates using replica exchange Monte Carlo simulations. For model building, they used the cryo-EM SARS-CoV-2 spike structure 6VSB for the open state [8]. The missing parts, such as the furin-cleavage loop, was modeled using CHARMMGUI [9].

For the validation of our structural generation procedure, we aligned and superposed with the algorithm of Needleman and Wunsch [10] our structure and the structure of Amaro *et al.* using the MOE software. Then, we determined the pairwise Gaussian-weighted RMSD (wRMSD) [11] of both structures with respect to each other. From the analysis of the superposed structures in Fig. 2S, we conclude that the structures deviate in some loop regions but otherwise agree well. This is confirmed by considering the wRMSD as a function of amino acid number in Fig. 3S and secondary structure as a function of amino acid number in Fig. 4S. We infer from these figures that almost all protein parts show values of wRMSD lower than 1 Å. Those regions, which have wRMSD values between 2-3 Å, are the S1 hinge (aa: 608-613) and RBD hinge (Asn317), visualized in Fig. 5S, which can easily relax through MD simulation. Since wRMSD values close to zero indicate high similarity between the structures [12], we conclude that our structure and the structure of Amaro *et al.* have a high structural similarity with minor differences in some coil regions.


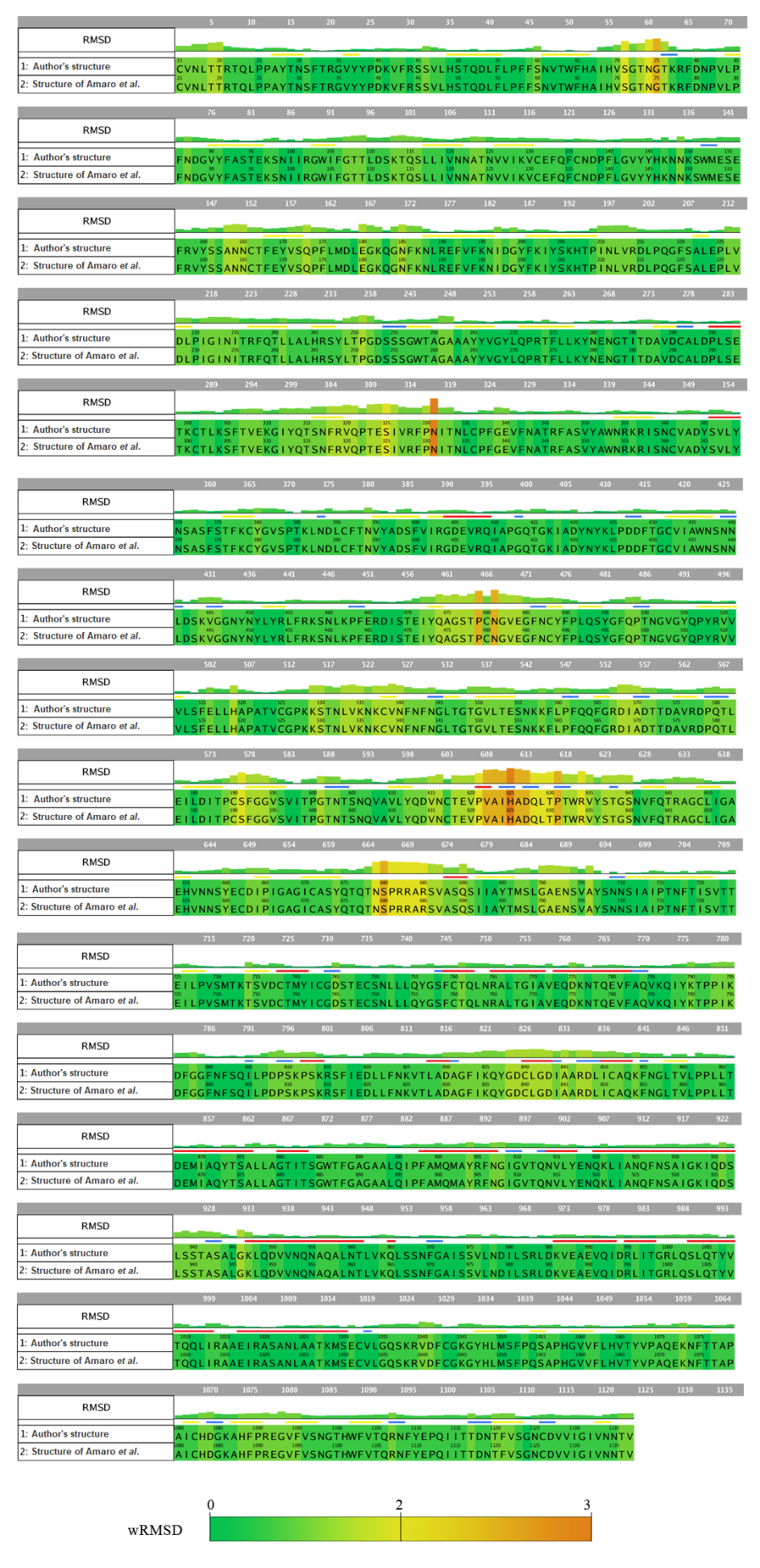


Fig. 3S: wRMSD of tertiary structures of the ACE2-bound S protein in the open state used as starting structures in our work and in the work of Amaro *et al*. as a function of amino acid number.


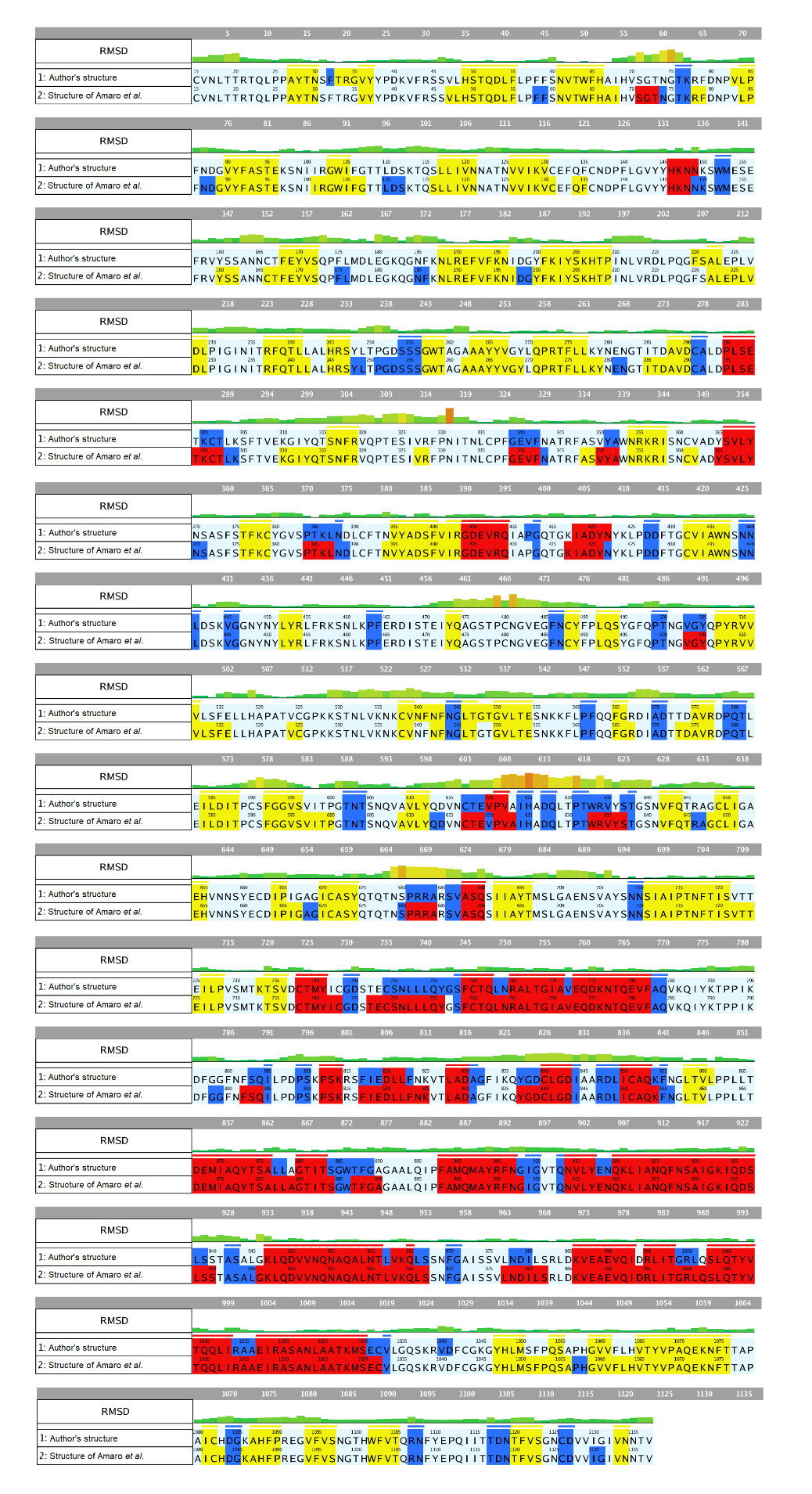


Fig. 4S: wRMSD of tertiary structures of the ACE2-bound S protein in the open state used as starting structures in our work and in the work of Amaro *et al*. as a function of amino acid number and secondary structure type [yellow: β-sheet, red: α-helix, blue: turn or coil].


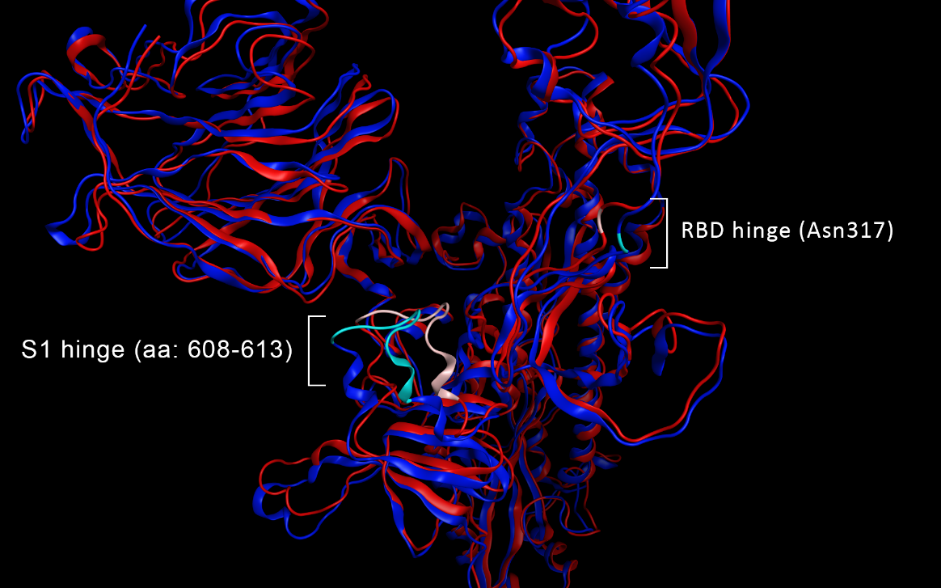


Fig. 5S: Tertiary structure superposition of our structure (red) with Amaro’s structure (blue). The regions of largest wRMSD values at the S1 hinge (aa: 608-613) and RBD hinge (Asn 317) are highlighted in colors [pink: our structure; cyan: Amaro‘s structure].

To further validate our modeled S1/S2-cleavage site, we compare it to the results of the work of Raghuvamsi *et al*. [13]. These authors performed amide hydrogen–deuterium exchange mass spectrometry in conjunction with MD simulations to investigate protein conformational dynamics and protein interactions of the S protein trimers. In their work they used an integrative model of full-length SARS-CoV-2 S protein obtained with the Modeller v.9.21 [3,4]. Their results revealed that the unstructured loop housing the S1/S2-cleavage site (aa: 677–689) is highly dynamic (Figure 2—figure supplement 4) with RMSF values reaching > 1.0 nm. Even though the cleavage site was abrogated in the construct used experimentally, they still observed increased dynamics at sites adjacent to the S1/S2-cleavage site, inferred by the higher relative deuterium exchange.

**S3. Structural generation procedure of the N-/O-glycosylated S protein**

Tab. S1: Glycan composition of chain A of N-/O-glycosylated S protein.

| Chain A | | | |
| --- | --- | --- | --- |
| Site | Type | Structure | Sequences |
| N17 | FA2 | 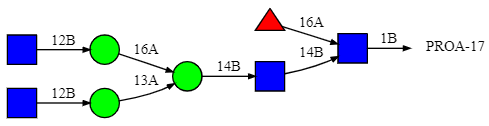 | bDGlcNAc(1→2)aDMan(1→6)[bDGlcNAc(1→2)aDMan(1→3)]bDMan(1→4)bDGlcNAc(1→4)[aLFuc(1→6)]bDGlcNAc(1→)PROA-17 |
| N61 | M5 | 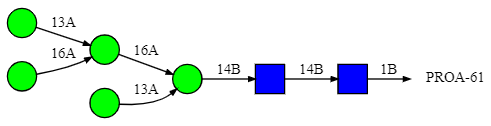 | aDMan(1→3)[aDMan(1→6)]aDMan(1→6)[aDMan(1→3)]bDMan(1→4)bDGlcNAc(1→4)bDGlcNAc(1→)PROA-61 |
| N74 | A3 | 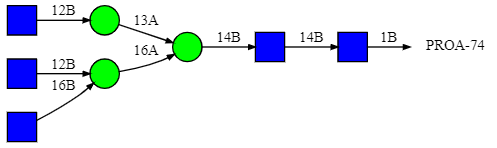 | bDGlcNAc(1→2)aDMan(1→3)[bDGlcNAc(1→2)[bDGlcNAc(1→6)]aDMan(1→6)]bDMan(1→4)bDGlcNAc(1→4)bDGlcNAc(1→)PROA-74 |
| N122 | M5 | 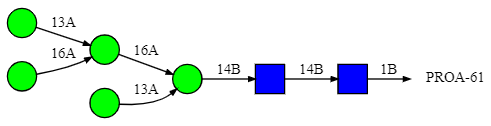 | aDMan(1→3)[aDMan(1→6)]aDMan(1→6)[aDMan(1→3)]bDMan(1→4)bDGlcNAc(1→4)bDGlcNAc(1→)PROA-122 |
| N149 | FA2G2S1 | 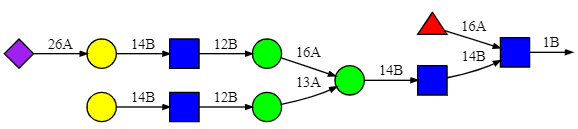 | aDNeu5Ac(2→6)bDGal(1→4)bDGlcNAc(1→2)aDMan(1→6)[bDGal(1→4)bDGlcNAc(1→2)aDMan(1→3)]bDMan(1→4)bDGlcNAc(1→4)[aLFuc(1→6)]bDGlcNAc(1→)PROA-149 |
| N165 | FA2G2S2 | 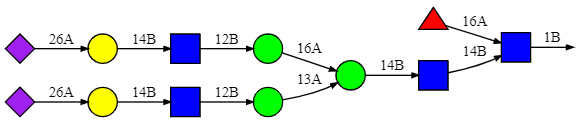 | aDNeu5Ac(2→6)bDGal(1→4)bDGlcNAc(1→2)aDMan(1→6)[aDNeu5Ac(2→6)bDGal(1→4)bDGlcNAc(1→2)aDMan(1→3)]bDMan(1→4)bDGlcNAc(1→4)[aLFuc(1→6)]bDGlcNAc(1→)PROA-165 |
| N234 | M8 | 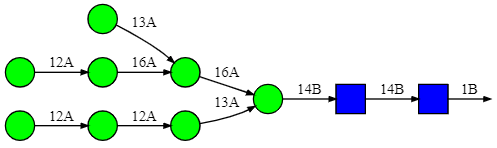 | aDMan(1→2)aDMan(1→6)[aDMan(1→3)]aDMan(1→6)[aDMan(1→2)aDMan(1→2)aDMan(1→3)]bDMan(1→4)bDGlcNAc(1→4)bDGlcNAc(1→)PROA-234 |
| N282 | FA3 | 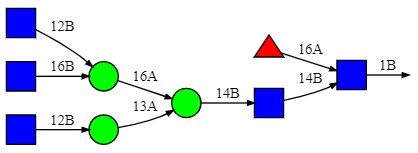 | bDGlcNAc(1→2)[bDGlcNAc(1→6)]aDMan(1→6)[bDGlcNAc(1→2)aDMan(1→3)]bDMan(1→4)bDGlcNAc(1→4)[aLFuc(1→6)]bDGlcNAc(1→)PROA-282 |
| N331 | FA2 | 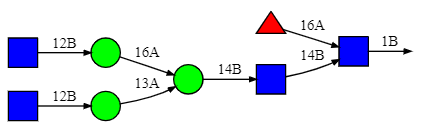 | bDGlcNAc(1→2)aDMan(1→6)[bDGlcNAc(1→2)aDMan(1→3)]bDMan(1→4)bDGlcNAc(1→4)[aLFuc(1→6)]bDGlcNAc(1→)PROA-331 |
| N343 | FA2 | 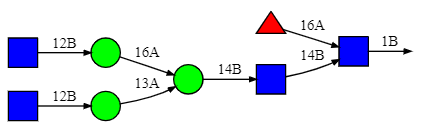 | bDGlcNAc(1→2)aDMan(1→6)[bDGlcNAc(1→2)aDMan(1→3)]bDMan(1→4)bDGlcNAc(1→4)[aLFuc(1→6)]bDGlcNAc(1→)PROA-343 |
| N603 | FA2 | 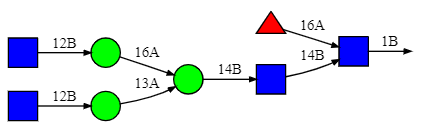 | bDGlcNAc(1→2)aDMan(1→6)[bDGlcNAc(1→2)aDMan(1→3)]bDMan(1→4)bDGlcNAc(1→4)[aLFuc(1→6)]bDGlcNAc(1→)PROA-603 |
| N616 | A2 | 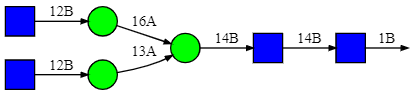 | bDGlcNAc(1→2)aDMan(1→6)[bDGlcNAc(1→2)aDMan(1→3)]bDMan(1→4)bDGlcNAc(1→4)bDGlcNAc(1→)PROA-616 |
| N657 | M5 | 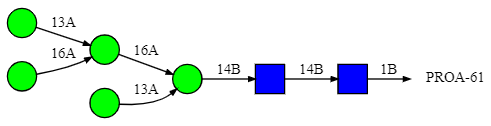 | aDMan(1→3)[aDMan(1→6)]aDMan(1→6)[aDMan(1→3)]bDMan(1→4)bDGlcNAc(1→4)bDGlcNAc(1→)PROA-657 |
| N709 | M6 | 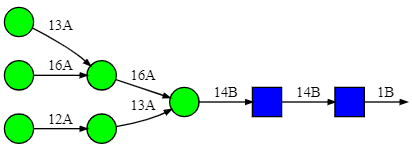 | aDMan(1→3)[aDMan(1→6)]aDMan(1→6)[aDMan(1→2)aDMan(1→3)]bDMan(1→4)bDGlcNAc(1→4)bDGlcNAc(1→)PROA-709 |
| N717 | HybridG1 | 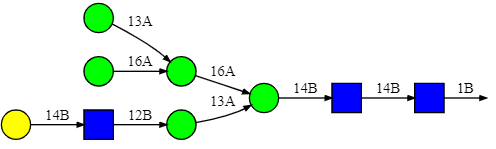 | bDGal(1→4)bDGlcNAc(1→2)aDMan(1→3)[aDMan(1→3)[aDMan(1→6)]aDMan(1→6)]bDMan(1→4)bDGlcNAc(1→4)bDGlcNAc(1→)PROA-717 |
| N801 | M6 | 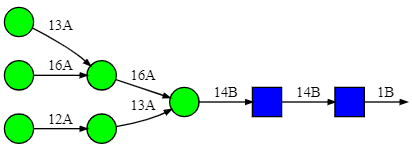 | aDMan(1→3)[aDMan(1→6)]aDMan(1→6)[aDMan(1→2)aDMan(1→3)]bDMan(1→4)bDGlcNAc(1→4)bDGlcNAc(1→)PROA-801 |
| N1074 | FA2G2S1 | 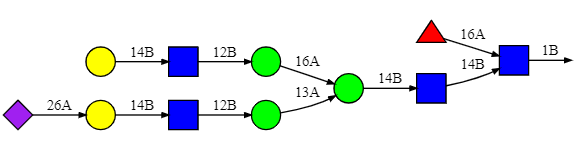 | aDNeu5Ac(2→6)bDGal(1→4)bDGlcNAc(1→2)aDMan(1→3)[bDGal(1→4)bDGlcNAc(1→2)aDMan(1→6)]bDMan(1→4)bDGlcNAc(1→4)[aLFuc(1→6)]bDGlcNAc(1→)PROA-1074 |
| N1098 | FA2 | 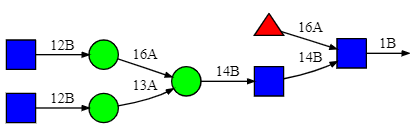 | bDGlcNAc(1→2)aDMan(1→6)[bDGlcNAc(1→2)aDMan(1→3)]bDMan(1→4)bDGlcNAc(1→4)[aLFuc(1→6)]bDGlcNAc(1→)PROA-1098 |
| N1134 | FA1 | 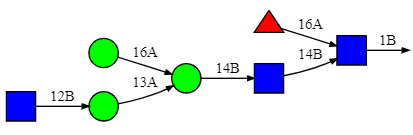 | bDGlcNAc(1→2)aDMan(1→3)[aDMan(1→6)]bDMan(1→4)bDGlcNAc(1→4)[aLFuc(1→6)]bDGlcNAc(1→)PROA-1134 |
| N1158 | A2 | 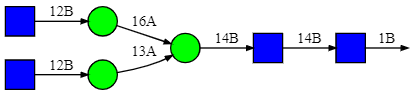 | bDGlcNAc(1→2)aDMan(1→6)[bDGlcNAc(1→2)aDMan(1→3)]bDMan(1→4)bDGlcNAc(1→4)bDGlcNAc(1→)PROA-1158 |
| N1173 | FA4 | 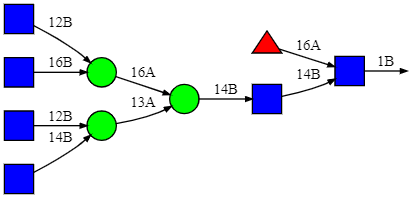 | bDGlcNAc(1→2)[bDGlcNAc(1→6)]aDMan(1→6)[bDGlcNAc(1→2)[bDGlcNAc(1→4)]aDMan(1→3)]bDMan(1→4)bDGlcNAc(1→4)[aLFuc(1→6)]bDGlcNAc(1→)PROA-1173 |
| N1194 | FA4G4S1 | 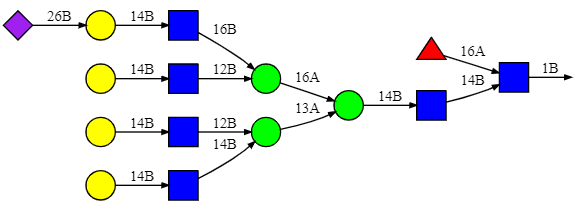 | bDNeu5Ac(2→6)bDGal(1→4)bDGlcNAc(1→6)[bDGal(1→4)bDGlcNAc(1→2)]aDMan(1→6)[bDGal(1→4)bDGlcNAc(1→2)[bDGal(1→4)bDGlcNAc(1→4)]aDMan(1→3)]bDMan(1→4)bDGlcNAc(1→4)[aLFuc(1→6)]bDGlcNAc(1→)PROA-1194 |
| T323 | O-glycan | 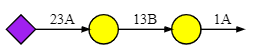 | aDNeu5Ac(2→3)bDGal(1→3)aDGal(1→)PROA-323 |
| S325 | O-glycan | 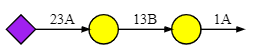 | aDNeu5Ac(2→3)bDGal(1→3)aDGal(1→)PROA-325 |

Tab. S2: Glycan composition of chain B of N-/O-glycosylated S protein.

| Chain B | | | |
| --- | --- | --- | --- |
| N17 | FA3 | 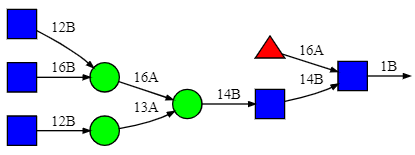 | \|  \| \| --- \| \| bDGlcNAc(1→6)[bDGlcNAc(1→2)]aDMan(1→6)[bDGlcNAc(1→2)aDMan(1→3)]bDMan(1→4)bDGlcNAc(1→4)[aLFuc(1→6)]bDGlcNAc(1→)PROB-17 \| |
| N61 | M5 | 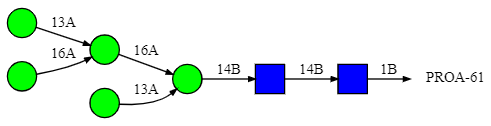 | aDMan(1→6)[aDMan(1→3)]aDMan(1→6)[aDMan(1→3)]bDMan(1→4)bDGlcNAc(1→4)bDGlcNAc(1→)PROB-61 |
| N74 | FA3G3S2 | 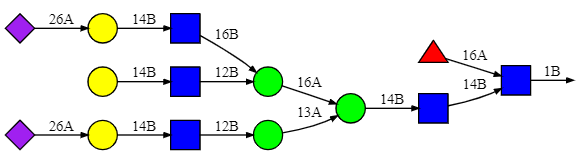 | aDNeu5Ac(2→6)bDGal(1→4)bDGlcNAc(1→6)[bDGal(1→4)bDGlcNAc(1→2)]aDMan(1→6)[aDNeu5Ac(2→6)bDGal(1→4)bDGlcNAc(1→2)aDMan(1→3)]bDMan(1→4)bDGlcNAc(1→4)[aLFuc(1→6)]bDGlcNAc(1→)PROB-74 |
| N122 | FA2 | 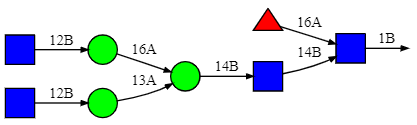 | bDGlcNAc(1→2)aDMan(1→6)[bDGlcNAc(1→2)aDMan(1→3)]bDMan(1→4)bDGlcNAc(1→4)[aLFuc(1→6)]bDGlcNAc(1→)PROB-122 |
| N149 | FA3 | 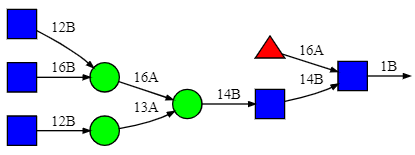 | bDGlcNAc(1→6)[bDGlcNAc(1→2)]aDMan(1→6)[bDGlcNAc(1→2)aDMan(1→3)]bDMan(1→4)bDGlcNAc(1→4)[aLFuc(1→6)]bDGlcNAc(1→)PROB-149 |
| N165 | M5 | 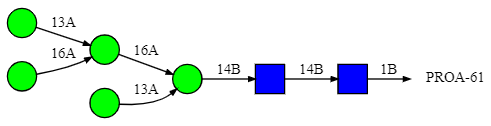 | aDMan(1→6)[aDMan(1→3)]aDMan(1→6)[aDMan(1→3)]bDMan(1→4)bDGlcNAc(1→4)bDGlcNAc(1→)PROB-165 |
| N234 | M9 | 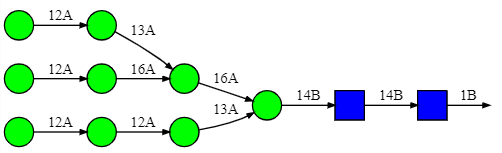 | aDMan(1→2)aDMan(1→6)[aDMan(1→2)aDMan(1→3)]aDMan(1→6)[aDMan(1→2)aDMan(1→2)aDMan(1→3)]bDMan(1→4)bDGlcNAc(1→4)bDGlcNAc(1→)PROB-234 |
| N282 | FA3G3S1 | 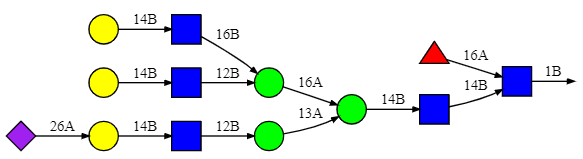 | aDNeu5Ac(2→6)bDGal(1→4)bDGlcNAc(1→2)aDMan(1→3)[bDGal(1→4)bDGlcNAc(1→6)[bDGal(1→4)bDGlcNAc(1→2)]aDMan(1→6)]bDMan(1→4)bDGlcNAc(1→4)[aLFuc(1→6)]bDGlcNAc(1→)PROB-282 |
| N331 | FA2 | 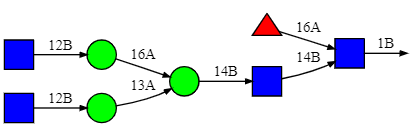 | bDGlcNAc(1→2)aDMan(1→6)[bDGlcNAc(1→2)aDMan(1→3)]bDMan(1→4)bDGlcNAc(1→4)[aLFuc(1→6)]bDGlcNAc(1→)PROB-331 |
| N343 | FA1 | 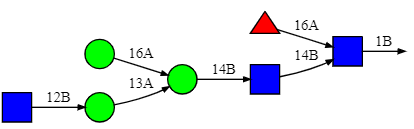 | bDGlcNAc(1→2)aDMan(1→3)[aDMan(1→6)]bDMan(1→4)bDGlcNAc(1→4)[aLFuc(1→6)]bDGlcNAc(1→)PROB-343 |
| N603 | M5 | 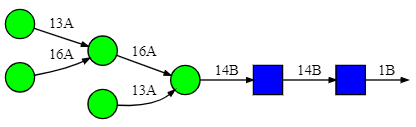 | aDMan(1→6)[aDMan(1→3)]aDMan(1→6)[aDMan(1→3)]bDMan(1→4)bDGlcNAc(1→4)bDGlcNAc(1→)PROB-603 |
| N616 | FA2 | 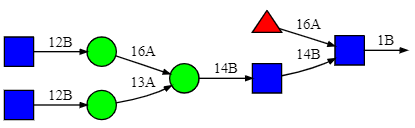 | bDGlcNAc(1→2)aDMan(1→6)[bDGlcNAc(1→2)aDMan(1→3)]bDMan(1→4)bDGlcNAc(1→4)[aLFuc(1→6)]bDGlcNAc(1→)PROB-616 |
| N657 | HybridG1 | 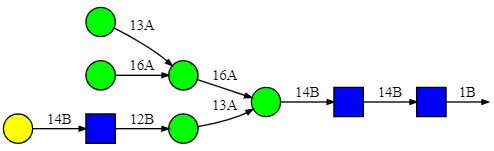 | bDGal(1→4)bDGlcNAc(1→2)aDMan(1→3)[aDMan(1→6)[aDMan(1→3)]aDMan(1→6)]bDMan(1→4)bDGlcNAc(1→4)bDGlcNAc(1→)PROB-657 |
| N709 | M5 | 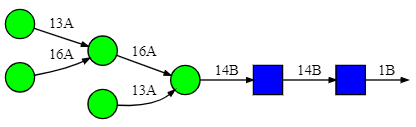 | aDMan(1→6)[aDMan(1→3)]aDMan(1→6)[aDMan(1→3)]bDMan(1→4)bDGlcNAc(1→4)bDGlcNAc(1→)PROB-709 |
| N717 | M5 | 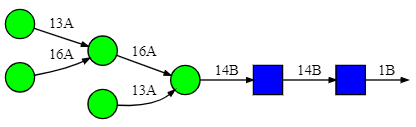 | aDMan(1→6)[aDMan(1→3)]aDMan(1→6)[aDMan(1→3)]bDMan(1→4)bDGlcNAc(1→4)bDGlcNAc(1→)PROB-717 |
| N801 | M7 | 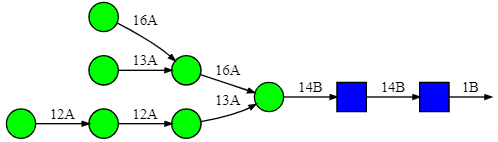 | aDMan(1→2)aDMan(1→2)aDMan(1→3)[aDMan(1→6)[aDMan(1→3)]aDMan(1→6)]bDMan(1→4)bDGlcNAc(1→4)bDGlcNAc(1→)PROB-801 |
| N1074 | M5 | 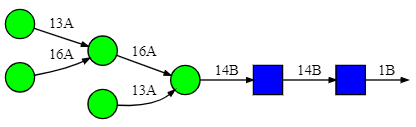 | aDMan(1→6)[aDMan(1→3)]aDMan(1→6)[aDMan(1→3)]bDMan(1→4)bDGlcNAc(1→4)bDGlcNAc(1→)PROB-1074 |
| N1098 | A2 | 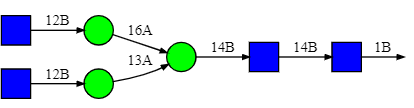 | bDGlcNAc(1→2)aDMan(1→6)[bDGlcNAc(1→2)aDMan(1→3)]bDMan(1→4)bDGlcNAc(1→4)bDGlcNAc(1→)PROB-1098 |
| N1134 | FA3 | 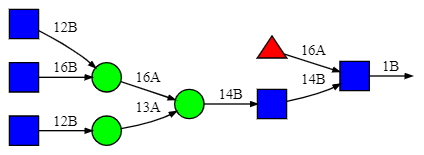 | bDGlcNAc(1→6)[bDGlcNAc(1→2)]aDMan(1→6)[bDGlcNAc(1→2)aDMan(1→3)]bDMan(1→4)bDGlcNAc(1→4)[aLFuc(1→6)]bDGlcNAc(1→)PROB-1134 |
| N1158 | FA2G2S1 | 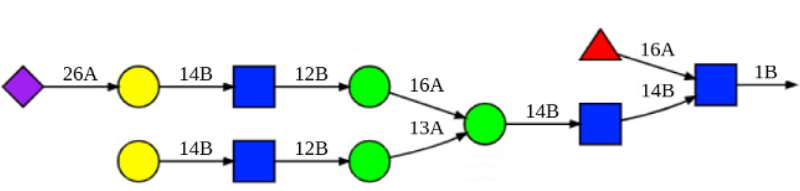 | aDNeu5Ac(2→6)bDGal(1→4)bDGlcNAc(1→2)aDMan(1→6)[bDGal(1→4)bDGlcNAc(1→2)aDMan(1→3)]bDMan(1→4)bDGlcNAc(1→4)[aLFuc(1→6)]bDGlcNAc(1→)PROB-1158 |
| N1173 | FA4 | 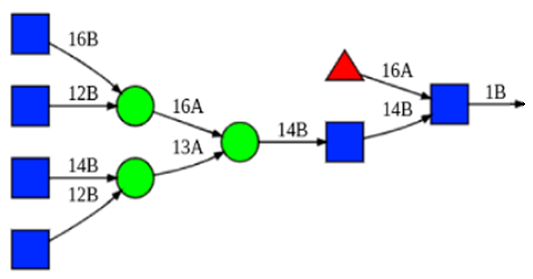 | bDGlcNAc(1→6)[bDGlcNAc(1→2)]aDMan(1→6)[bDGlcNAc(1→4)[bDGlcNAc(1→2)]aDMan(1→3)]bDMan(1→4)bDGlcNAc(1→4)[aLFuc(1→6)]bDGlcNAc(1→)PROB-1173 |
| N1194 | FA4G4S1 | 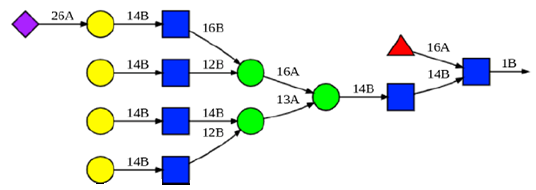 | aDNeu5Ac(2→6)bDGal(1→4)bDGlcNAc(1→6)[bDGal(1→4)bDGlcNAc(1→2)]aDMan(1→6)[bDGal(1→4)bDGlcNAc(1→4)[bDGal(1→4)bDGlcNAc(1→2)]aDMan(1→3)]bDMan(1→4)bDGlcNAc(1→4)[aLFuc(1→6)]bDGlcNAc(1→)PROB-1194 |
| T323 | O-glycan | 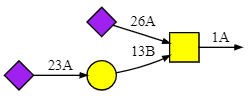 | aDNeu5Ac(2→3)bDGal(1→3)[aDNeu5Ac(2→6)]aDGalNAc(1→)PROB-323 |

Tab. S3: Glycan composition of chain C of N-/O-glycosylated S protein.

| Chain C | | | |
| --- | --- | --- | --- |
| N17 | FA3 | 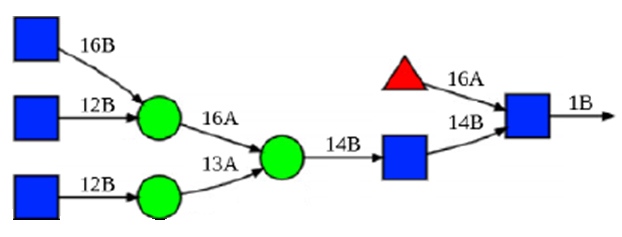 | bDGlcNAc(1→6)[bDGlcNAc(1→2)]aDMan(1→6)[bDGlcNAc(1→2)aDMan(1→3)]bDMan(1→4)bDGlcNAc(1→4)[aLFuc(1→6)]bDGlcNAc(1→)PROC-17 |
| N61 | M5 | 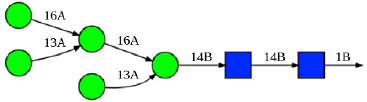 | aDMan(1→6)[aDMan(1→3)]aDMan(1→6)[aDMan(1→3)]bDMan(1→4)bDGlcNAc(1→4)bDGlcNAc(1→)PROC-61 |
| N74 | A2 | 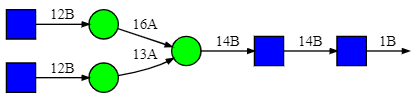 | bDGlcNAc(1→2)aDMan(1→6)[bDGlcNAc(1→2)aDMan(1→3)]bDMan(1→4)bDGlcNAc(1→4)bDGlcNAc(1→)PROC-74 |
| N122 | M5 | 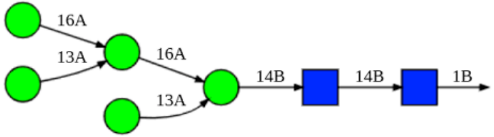 | aDMan(1→6)[aDMan(1→3)]aDMan(1→6)[aDMan(1→3)]bDMan(1→4)bDGlcNAc(1→4)bDGlcNAc(1→)PROC-122 |
| N149 | FA2 | 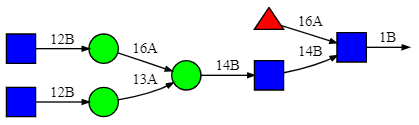 | bDGlcNAc(1→2)aDMan(1→6)[bDGlcNAc(1→2)aDMan(1→3)]bDMan(1→4)bDGlcNAc(1→4)[aLFuc(1→6)]bDGlcNAc(1→)PROC-149 |
| N165 | FA2G2S1 | 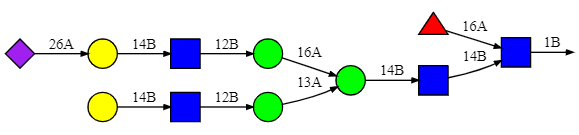 | aDNeu5Ac(2→6)bDGal(1→4)bDGlcNAc(1→2)aDMan(1→6)[bDGal(1→4)bDGlcNAc(1→2)aDMan(1→3)]bDMan(1→4)bDGlcNAc(1→4)[aLFuc(1→6)]bDGlcNAc(1→)PROC-165 |
| N234 | M9 | 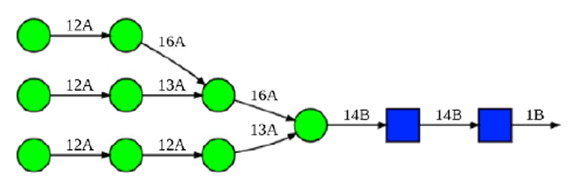 | aDMan(1→2)aDMan(1→6)[aDMan(1→2)aDMan(1→3)]aDMan(1→6)[aDMan(1→2)aDMan(1→2)aDMan(1→3)]bDMan(1→4)bDGlcNAc(1→4)bDGlcNAc(1→)PROC-234 |
| N282 | A2 | 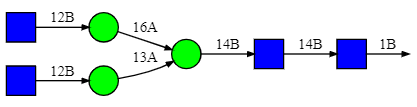 | bDGlcNAc(1→2)aDMan(1→6)[bDGlcNAc(1→2)aDMan(1→3)]bDMan(1→4)bDGlcNAc(1→4)bDGlcNAc(1→)PROC-282 |
| N331 | FA3G3S1 | 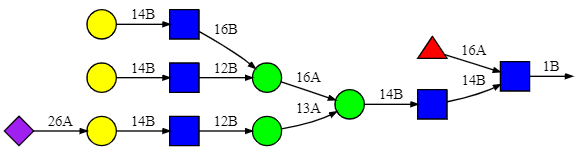 | aDNeu5Ac(2→6)bDGal(1→4)bDGlcNAc(1→2)aDMan(1→3)[bDGal(1→4)bDGlcNAc(1→6)[bDGal(1→4)bDGlcNAc(1→2)]aDMan(1→6)]bDMan(1→4)bDGlcNAc(1→4)[aLFuc(1→6)]bDGlcNAc(1→)PROC-331 |
| N343 | FA2 | 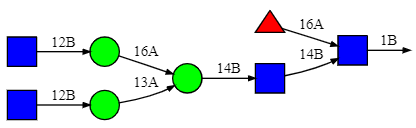 | bDGlcNAc(1→2)aDMan(1→6)[bDGlcNAc(1→2)aDMan(1→3)]bDMan(1→4)bDGlcNAc(1→4)[aLFuc(1→6)]bDGlcNAc(1→)PROC-343 |
| N603 | M5 | 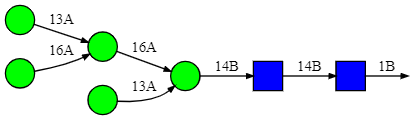 | aDMan(1→6)[aDMan(1→3)]aDMan(1→6)[aDMan(1→3)]bDMan(1→4)bDGlcNAc(1→4)bDGlcNAc(1→)PROC-603 |
| N616 | FA2 | 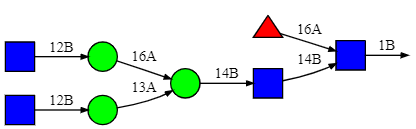 | bDGlcNAc(1→2)aDMan(1→6)[bDGlcNAc(1→2)aDMan(1→3)]bDMan(1→4)bDGlcNAc(1→4)[aLFuc(1→6)]bDGlcNAc(1→)PROC-616 |
| N657 | HybridG1 | 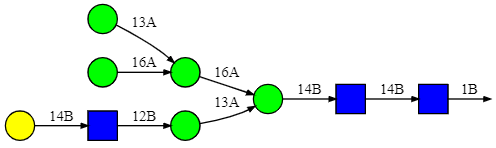 | bDGal(1→4)bDGlcNAc(1→2)aDMan(1→3)[aDMan(1→6)[aDMan(1→3)]aDMan(1→6)]bDMan(1→4)bDGlcNAc(1→4)bDGlcNAc(1→)PROC-657 |
| N709 | M5 | 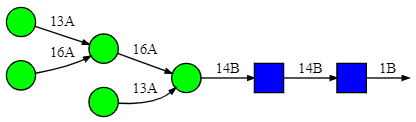 | aDMan(1→6)[aDMan(1→3)]aDMan(1→6)[aDMan(1→3)]bDMan(1→4)bDGlcNAc(1→4)bDGlcNAc(1→)PROC-709 |
| N717 | M6 | 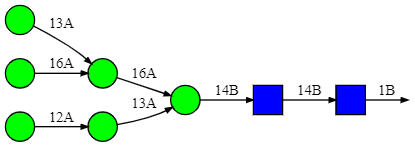 | aDMan(1→6)[aDMan(1→3)]aDMan(1→6)[aDMan(1→2)aDMan(1→3)]bDMan(1→4)bDGlcNAc(1→4)bDGlcNAc(1→)PROC-717 |
| N801 | M5 | 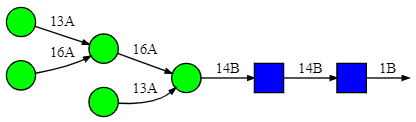 | aDMan(1→6)[aDMan(1→3)]aDMan(1→6)[aDMan(1→3)]bDMan(1→4)bDGlcNAc(1→4)bDGlcNAc(1→)PROC-801 |
| N1074 | M5 | 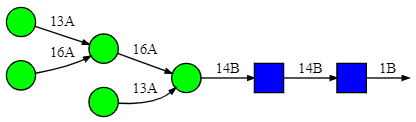 | aDMan(1→6)[aDMan(1→3)]aDMan(1→6)[aDMan(1→3)]bDMan(1→4)bDGlcNAc(1→4)bDGlcNAc(1→)PROC-1074 |
| N1098 | HybridG1S1 | 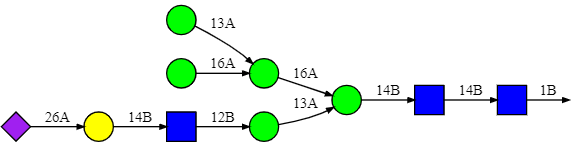 | aDNeu5Ac(2→6)bDGal(1→4)bDGlcNAc(1→2)aDMan(1→3)[aDMan(1→6)[aDMan(1→3)]aDMan(1→6)]bDMan(1→4)bDGlcNAc(1→4)bDGlcNAc(1→)PROC-1098 |
| N1134 | FA2 | 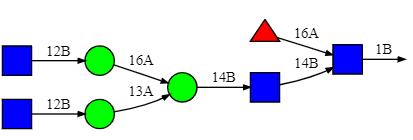 | bDGlcNAc(1→2)aDMan(1→6)[bDGlcNAc(1→2)aDMan(1→3)]bDMan(1→4)bDGlcNAc(1→4)[aLFuc(1→6)]bDGlcNAc(1→)PROC-1134 |
| N1158 | A2 |  | bDGlcNAc(1→2)aDMan(1→6)[bDGlcNAc(1→2)aDMan(1→3)]bDMan(1→4)bDGlcNAc(1→4)bDGlcNAc(1→)PROC-1158 |
| N1173 | FA4 |  | bDGlcNAc(1→6)[bDGlcNAc(1→2)]aDMan(1→6)[bDGlcNAc(1→4)[bDGlcNAc(1→2)]aDMan(1→3)]bDMan(1→4)bDGlcNAc(1→4)[aLFuc(1→6)]bDGlcNAc(1→)PROC-1173 |
| N1194 | FA4G4S1 |  | aDNeu5Ac(2→6)bDGal(1→4)bDGlcNAc(1→6)[bDGal(1→4)bDGlcNAc(1→2)]aDMan(1→6)[bDGal(1→4)bDGlcNAc(1→4)[bDGal(1→4)bDGlcNAc(1→2)]aDMan(1→3)]bDMan(1→4)bDGlcNAc(1→4)[aLFuc(1→6)]bDGlcNAc(1→)PROC-1194 |
| T323 | O-glycan | 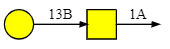 | bDGal(1→3)aDGalNAc(1→)PROC-323 |


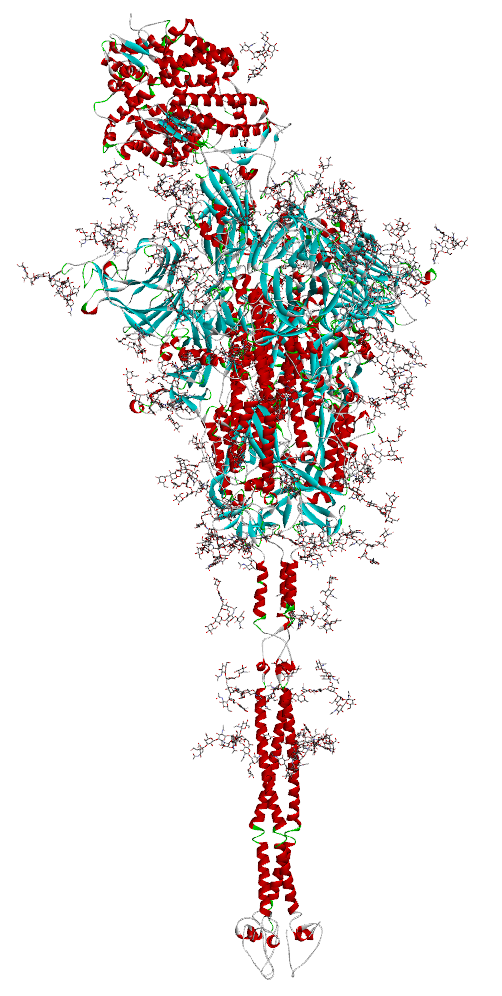


Fig. 6S: Tertiary structure of the full-length S protein in complex with ACE2 and O- as well as N-glycans, obtained with the Glycan Reader and Modeler tool implemented within CHARMM-GUI [14].

**S4. Additional details about MD-simulation phases**

| System | Simulation phase | Input from  simulation phase | Simulation time  [ns] |  |
| --- | --- | --- | --- | --- |
|  |  |  |  |  |
| S protein, closed state, 310 K | A |  | 50 |  |
| S protein, open state + ACE2, 310 K | A |  | 50 |  |
| S protein, open state + ACE2, 314 K | B |  | 50 |  |
| uncleaved S protein, open state + ACE2 + furin, 314 K | C | B | 30 |  |
| uncleaved S protein, open state + furin, 314 K | C1 | B | 30 |  |
| furin-cleavage-site-deleted S protein, 314 K | C2 | B | 30 |  |
| uncleaved S protein, open state + ACE2 + furin + TMPRSS2, 314 K | D | C | 30 |  |
| furin- and TMPRSS2-cleaved S protein, open state + ACE2 + furin + TMPRSS2, 314 K | E | D | 30 |  |
| N-/O-glycosylated S protein, open state + ACE2 + furin + TMPRSS2, 314 K | F |  | 300 |  |

Tab. S4: Information about input structures and simulation times of MD-simulation phases in our work.

**S5. Evidence for furin cleavage at S1/S2 of the S protein on the cell surface**

Experimental evidence that furin cleavage at S1/S2 of the S protein can also take place efficiently on the cell surface, expecially in *in vivo* systems, is discussed in detail in the following.

First of all, ACE2, TMPRSS2 and furin were found to be expressed in the nose and olfactory bulb in mice and humans, e.g. respiratory mucosa, cilia and cytoplasm of respiratory epithelial cells, olfactory mucosa [15], as well as the saliva of the oral cavity [16]. Since it has been found that the entry of SARS-CoV-2 primarily takes place in the nasal or oral cavity [17] with higher viral loads detected in the nose than in the oral cavity [18], the presence of ACE2, TMPRSS2, and furin in promixity of the SARS-CoV-2 spike is the most likely situation found during SARS-CoV-2 infection in *in vivo* systems.

Secondly, although it is generally believed that furin is membrane-bound localized in the Golgi apparatus, the study of Vidricaire *et al.* has demonstrated that there is also an active isoform of furin that can be secreted into the surrounding of host cells, which may facilitate the cleavage of the spike protein of SARS-CoV-2 at the cell surface [19-21]. They reported the presence of a secreted form of furin in the cellular neighborhood of cells infected with a vaccinia virus recombinant containing the furin gene. These results are supported by later works, which demonstrated that furin can be transported from the cell interior to cell surface and can also cleave proteins [22]. Furthermore, it has been demonstrated by Arsenault *et al.* [23] that relocalization of furin from the trans-Golgi network to endosomomal compartments and the cell surface can take place in case of cancer cells affected by hypoxia. Moreover, it has been shown that hypoxia downregulates SARS-CoV-2 receptors ACE2 and TMPRSS2 and inhibits viral entry [24]. Thus, we conclude from these works that in case of low levels of ACE2 and TMPRSS2, SARS-CoV-2 might use the furin cleavage process to increase the efficiency of viral entry and cell-cell fusion. In this case ACE2 binding and the resulting enhancement of furin cleavage S1/S2 as well as promotion of TMPRSS2 cleavage becomes more important, since cells with low levels of ACE2 and TMPRSS2 need to become more efficient to support TMPRSS2-dependent viral entry and cell-cell fusion, as demonstrated in our paper and supported by the work of Papa *et al.* [25].

**SI References**

[1] Molecular Operating Environment (MOE), Software version: 2019.0102, Chemical Computing Group ULC, 1010 Sherbooke St. West, Suite #910, Montreal, QC, Canada, H3A 2R7, 2022; https://www.chemcomp.com/Products.htm

[2] Casalino L, et al. (2020) Beyond Shielding: The Roles of Glycans in the SARS-CoV-2 Spike Protein. ACS Cent Sci 6:1722-1734

[3] Šali A, Blundell TL (1993) Comparative Protein Modelling by Satisfaction of Spatial Restraints. J Mol Biol 234:779-815; https://salilab.org/modeller/

[4] Webb B, Sali A (2016) Comparative Protein Structure Modeling Using MODELLER. Curr Protoc Bioinformatics 54:5.6.1-5.6.37

[5] Yang J, et al. (2015) The I-TASSER Suite: Protein structure and function prediction. Nat Methods 12:7-8

[6] Roy A, Kucukural A, Zhang Y (2010) I-TASSER: a unified platform for automated protein structure and function prediction. Nat Protocols 5:725-738

[7] Zhang Y (2008) I-TASSER server for protein 3D structure prediction. BMC Bioinformatics 9:40; https://zhanggroup.org/I-TASSER/

[8] PDB ID: 6VSB. Wrapp D, et al. (2020) Cryo-EM structure of the 2019-nCoV spike in the prefusion conformation. Science 367:1260-1263

[9] Jo S, Kim T, Iyer VG, Im W (2008) CHARMM-GUI: A Web-based Graphical User Interface for CHARMM. J Comput Chem 29:1859-1865; https://www.charmm-gui.org/

[10] Needleman SB, Wunsch CD (1970) A general method applicable to the search for similarities in the amino acid sequences of two proteins. J Mol Biol 48:443-453

[11] Damm KL, Carlson HA (2006) Gaussian-weighted RMSD superposition of proteins: a structural comparison for flexible proteins and predicted protein structures. Biophys J 90:4558-4573

[12] Reva BA, Finkelstein AV, Skolnick J (1998) What is the probability of a chance prediction of a protein structure with an rmsd of 6 Å ? Fold Des 3:141-147

[13] Raghuvamsi PV, et al. (2021) SARS-CoV-2 S protein: ACE2 interaction reveals novel allosteric targets. eLife 10:e63646

[14] Park SJ, et al. (2019) CHARMM-GUI Glycan Modeler for modeling and simulation of carbohydrates and glycoconjugates. Glycobiology 29:320-331

[15] Ueha R, et al. (2021) ACE2, TMPRSS2, and Furin expression in the nose and olfactory bulb in mice and humans. Rhinology 59:105-109

[16] Sakaguchi W, et al. (2020) Existence of SARS-CoV-2 Entry Molecules in the Oral Cavity. Int J Mol Sci 21:6000

[17] Gallo O, Locatello LG, Mazzoni A, Novelli L, Annunziato F (2021) The central role of the nasal microenvironment in the transmission, modulation, and clinical progression of SARS-CoV-2 infection. Mucosal Immunol 14:305-316

[18] Zhou L, et al. (2020) SARS-CoV-2 viral load in upper respiratory specimens of infected patients. N Engl J Med 382:12

[19] Vidricaire G, Denault JB, Leduc R (1993) Characterization of a Secreted Form of Human Furin Endoprotease. Biochem Biophys Res Commun 195:1011-1018

[20] Zhang Y, Tang LV (2021) Overview of Targets and Potential Drugs of SARS-CoV-2 According to the Viral Replication. J Proteome Res 20:49-59

[21] Lukassen S, et al. (2020) SARS‐CoV‐2 receptor ACE2 and TMPRSS2 are primarily expressed in bronchial transient secretory cells. EMBO J 39:e105114

[22] Molloy SS, Thomas L, VanSlyke JK, Stenberg PE, Thomas G (1994) Intracellular trafficking and activation of the furin proprotein convertase: localization to the TGN and recycling from the cell surface. EMBO J 13:18-33

[23] Arsenault D, Lucien F, Dubois CM (2012) Hypoxia enhances cancer cell invasion through relocalization of the proprotein convertase furin from the trans-Golgi network to the cell surface, J Cell Physiol 227:789-800

[24] Wing PAC, et al. (2021) Hypoxic and pharmacological activation of HIF inhibits SARS-CoV-2 infection of lung epithelial cells. Cell Rep 35:109020

[25] Papa G, et al. (2021) Furin cleavage of SARS-CoV-2 Spike promotes but is not essential for infection and cell-cell fusion. PLoS Pathog 17:e1009246
